# Efficacy and potential determinants of exercise therapy in knee and hip osteoarthritis: A systematic review and meta-analysis

**DOI:** 10.1016/j.rehab.2019.04.006

**Published:** 2019-09

**Authors:** Siew-Li Goh, Monica S.M. Persson, Joanne Stocks, Yunfei Hou, Jianhao Lin, Michelle C. Hall, Michael Doherty, Weiya Zhang

**Affiliations:** a Arthritis Research UK Pain Centre, Academic Rheumatology, University of Nottingham, Clinical Sciences Building, City Hospital, NG5 1PB Nottingham, UK; b Sports Medicine Unit, University of Malaya, 50603 Kuala Lumpur, Malaysia; c Arthritis Clinical and Research Centre, Peking University People's Hospital, Beijing, China; d Division of Physiotherapy Rehabilitation Sciences Education, University of Nottingham, NG5 1PB Nottingham, UK

**Keywords:** Knee osteoarthritis, Exercise, Meta-analysis, Determinants, Physical therapy, Pain, Function, Quality of Life

## Abstract

**Background:**

Exercise is an effective treatment for osteoarthritis. However, the effect may vary from one patient (or study) to another.

**Objective:**

To evaluate the efficacy of exercise and its potential determinants for pain, function, performance, and quality of life (QoL) in knee and hip osteoarthritis (OA).

**Methods:**

We searched 9 electronic databases (AMED, CENTRAL, CINAHL, EMBASE, MEDLINE Ovid, PEDro, PubMed, SPORTDiscus and Google Scholar) for reports of randomised controlled trials (RCTs) comparing exercise-only interventions with usual care. The search was performed from inception up to December 2017 with no language restriction. The effect size (ES), with its 95% confidence interval (CI), was calculated on the basis of between-group standardised mean differences. The primary endpoint was at or nearest to 8 weeks. Other outcome time points were grouped into intervals, from < 1 month to ≥ 18 months, for time-dependent effects analysis. Potential determinants were explored by subgroup analyses. Level of significance was set at *P* ≤ 0.10.

**Results:**

Data from 77 RCTs (6472 participants) confirmed statistically significant exercise benefits for pain (ES 0.56, 95% CI 0.44–0.68), function (0.50, 0.38–0.63), performance (0.46, 0.35–0.57), and QoL (0.21, 0.11–0.31) at or nearest to 8 weeks. Across all outcomes, the effects appeared to peak around 2 months and then gradually decreased and became no better than usual care after 9 months. Better pain relief was reported by trials investigating participants who were younger (mean age < 60 years), had knee OA, and were not awaiting joint replacement surgery.

**Conclusions:**

Exercise significantly reduces pain and improves function, performance and QoL in people with knee and hip OA as compared with usual care at 8 weeks. The effects are maximal around 2 months and thereafter slowly diminish, being no better than usual care at 9 to 18 months. Participants with younger age, knee OA and not awaiting joint replacement may benefit more from exercise therapy. These potential determinants, identified by study-level analyses, may have implied ecological bias and need to be confirmed with individual patient data.

## 1 Introduction

Osteoarthritis (OA) of the lower limb is a common joint condition affecting older people, with approximately 10% to 20% of people ≥ 60 years old worldwide experiencing pain in knees [1]. It is associated with considerable individual and societal healthcare burden [2] and accounts for 80% to 90% of hip and knee replacements in the United States and United Kingdom [3], [4], [5]. International guidelines for managing OA recommend exercise as a core non-pharmacological therapy. Exercise improves symptoms and the general well-being of people while being relatively safe as compared with pharmacological treatments [6].

Improved pain and functional outcomes after exercise therapy in OA are well demonstrated by numerous meta-analyses [7], [8]. However, some gaps in evidence still exist. First, evidence for the benefits of exercise therapy for outcomes other than pain and self-reported function such as quality of life (QoL), muscle strength, or task performance are still inconclusive [9]. Second, predictors of treatment response for exercise therapy in OA have yet to be explored.

This systematic review and meta-analysis aimed to comprehensively review the efficacy of exercise therapy for pain, function, performance, and QoL. In addition, it aimed to preliminarily explore potential determinants of exercise therapy in knee and hip OA and to assess whether the effect of exercise is robust to heterogeneous studies.

## 2 Methods

This systematic review and meta-analysis is part of a project with a broader scope, primarily aiming to estimate the relative efficacy of different types of exercise. The protocol for the wider project has been registered (PROSPERO CRD42016033865) and published [10].

### 2.1 Search strategy

The following electronic bibliographic databases were systematically searched from dates of inception to December 2015: Allied and Complementary Medicine Database (AMED), The Cochrane Central Register of Controlled Trials (CENTRAL), Cumulative Index to Nursing and Allied Health Literature (CINAHL), Excerpta Medica Database (EMBASE), MEDLINE Ovid, Physiotherapy Evidence Database (PEDro), PubMed, SPORTDiscus, and Google Scholar. The literature search was updated in December 2017. Reports of randomised controlled trials (RCTs) in any language were included as long as trials compared an exercise intervention to a non-exercise intervention or to another type of exercise in knee and hip OA. An example of the MEDLINE search is in Appendix 1. Title, abstract and full text screening was performed by one reviewer (SLG), validated by a second reviewer (MH). A third reviewer (WZ) was involved if any discrepancies arose. A similar process was applied for data extraction with MSMP, JS, YFH and WZ involved in validations.

### 2.2 Inclusion criteria

In addition to fulfilling the broader search criteria mentioned above, RCTs also had to fulfill the following criteria to be included in the meta-analysis:

• participants had knee or hip OA and had not undergone knee or hip joint replacement surgery;
• exercise-only interventions (regardless of exercise type) without additional treatment were examined;
• control groups were assigned to usual care (i.e., controls receiving no new interventions, including those assigned to continue their usual physician follow-up, usual physical activity, or on a “waiting list” for which the active intervention would be offered only after the study period);
• reporting of pain, function, performance, or QoL outcomes.

### 2.3 Outcomes

The primary outcome was pain, and secondary outcomes were self-reported function, objective performance, and QoL. If more than one scale was reported for an outcome, we selected the scale that was more comprehensively reported and highest in the ranking order proposed by Fransen [11] and Regnaux [12].

For performance measures, gait/walking parameters (e.g., walking distance, walking time etc.) were given priority because measurement and reporting of these parameters were relatively standard across trials as compared with other performance outcomes such as strength/power. Joint-specific parameters, such as strength, power, and range of motion, were used only if gait parameters were not available. Strength parameters were in order of preference from knee extensors, knee flexors, hip abductors, and other muscle groups. When tests performed at varying intensity were reported, the results from the highest intensity were chosen.

Because of no consensus or evidence to support which of these physical measures should be the gold standard for assessment in physical improvements in OA, we believe that any physical measures used/reported by the authors should be given consideration. Arguably, these measures are clinically different, but in many instances, a battery of physical tests (i.e., combination of different parameters and not just one specific measure) are recommended for physical assessment of individuals with OA [13], [14].

Furthermore, this meta-analysis adjusted between-measurement differences (by standardizing the difference in means to the variance of the group measures) before pooling for analysis, just as different measures of pain with different pain scales were standardised according to the SD of the measure. This procedure allowed us to pool the different scales together.

### 2.4 Study time points

Outcomes reported at different durations of follow-up were recorded. The primary time point was chosen at or nearest to 8 weeks after baseline/randomisation because this was the most common point reported. Additionally, the time points were grouped into intervals (i.e., < 1 month, ≥ 1 month, and ≥ 2, ≥ 3, ≥ 6, ≥ 9, ≥ 12, and ≥ 18 months) to examine time-dependent effects. For exploring time dependent effects, the analysis was arbitrarily limited to studies with effect size (ES) < 2 to minimise bias due to outlying studies. If the number of studies for a particular time point was small, such outlying data would contribute to a disproportionately large estimate.

### 2.5 Handling of multi-arm studies

For studies with > 1 eligible exercise arm and a shared usual care comparator, only one exercise arm was analysed to avoid double-counting the participants in the usual care group [15]. Such studies were handled as follows: 1) if the types of exercise differed, the exercise that was less frequently investigated by RCTs was chosen and 2) if the exercises were similar, the exercise intervention groups were aggregated for analysis. For example, if an RCT had 4 groups — group 1 (supervised Tai-chi), group 2 (unsupervised Tai-chi), group 3 (strengthening exercise), and group 4 (usual care) — the Tai-chi exercise groups would be selected for analysis above the strengthening group because Tai-chi trials are comparatively scarce in the literature. Because groups 1 and 2 are similar exercise types, data from these 2 groups would be aggregated before being compared to usual care. In some cases, one study may provide 2 sets of ESs such as when outcomes for knee and hip OA or for males and females were reported separately. Details of the calculations and method of aggregation are in the published protocol [10].

### 2.6 Calculation of ES and SD

The mean change score (mean end-point minus mean baseline score) was calculated for each group. The SD of the change was calculated as SD = √(SD_0_^2^ + SD_1_^2^ - 2 × r × SD_0_ × SD_1_) [16], where r is the correlation coefficient between baseline and endpoint outcome scores. We assumed r to be 0.5 in this analysis. The ES was calculated by using the between-group standardised mean difference (SMD) following Cohen's method [16]. With this definition, an ES 0.2, 0.5, and 0.8 is considered small, moderate or large, respectively [17]. We attempted to contact authors for missing data but were rarely successful. If the change score could not be extracted or derived, the mean endpoint score was used instead. If a report did not give the within-group SD or did not provide sufficient information to calculate it, the missing SD was imputed from other studies. The missing SD was imputed by using the largest SD of the same scale reported for other trials if available; otherwise an arithmetic mean of other SDs was used [18]. A random effects model was used to pool the data. Data analysis involved using Stata (StataCorp. 2017. Stata Statistical Software: Release 15. College Station, TX).

### 2.7 Determinants

Subgroup analyses were performed to identify potential determinants of efficacy according to the following study characteristics that were commonly reported in exercise trials:

• Clinical characteristics:

∘ mean age – < 60 or ≥ 60 years (dichotomised according to the international guideline of aging population [19]),
∘ mean body mass index (BMI) – < 30 or ≥ 30 kg/m^2^, which corresponds to a classification of obesity [20],
∘ percentage of female participants arbitrarily divided into < 60%, 60% to 80% or ≥ 80%,
∘ joint sites (knee OA, hip OA, or mixed knee and hip OA),
∘ whether or not participants were recruited from waiting lists for joint surgery.

• Methodological characteristics:

∘ pain criteria (whether a pain severity threshold was set as inclusion criteria),
∘ American College of Rheumatology (ACR) criteria (whether recruitment was based on ACR criteria),
∘ radiographic criteria (whether radiographic changes were required for recruitment),
∘ recruitment setting (specialist/hospital cohort, general practitioner [GP]/community, mixed cohort). Studies with unclear recruitment were excluded from subgroup analysis,
∘ whether adherence was monitored,
∘ whether monitoring or control for pharmacological analgesics was reported,
∘ whether intention-to-treat (ITT) analysis was undertaken,
∘ whether the study had > 100 participants per arm,
∘ whether walking parameters or other physical test parameters were used to measure performance,
∘ whether a disease-specific or generic tool was used to assess QoL changes.

These variables were selected for their potential as effect modifiers of exercise response (e.g., joint site, mean age) or as sources of bias (e.g., adherence, ITT analysis, sample size).

### 2.8 Meta-regression

Any potential covariates of the effect of exercise with *P* ≤ 0.10 identified on univariate meta-regression were subsequently included in multivariate meta-regression modelling. The level of significance for multivariate meta-regression was also set at *P* ≤ 0.10 [21].

### 2.9 Sensitivity analysis

Analyses were repeated in the following ways to assess whether the results were robust:

1. using scores at end points instead of change scores;
2. including outliers;
3. excluding studies that had imputed SD;
4. excluding studies that assessed 2, rather than 1, index knee in each participant;
5. excluding translated publications; and
6. excluding studies with high heterogeneity as assessed by Baujat plots [22].

The Baujat plot is useful for visually assessing the source of heterogeneity because studies with high contributions to heterogeneity and the overall pooled estimate can be identified. These studies were excluded sequentially until the I^2^ statistic (an indicator of unexplained heterogeneity) was < 30%. A level of 30% suggests unremarkable influence of subgroup diversity, 30% to 60% suggests moderate heterogeneity, 50% to 90% substantial heterogeneity, and 75% to 100% considerable heterogeneity [16], [23].

### 2.10 Risk of bias assessment

A modified Cochrane risk of bias assessment tool was used to assess the quality of studies [24]. Various sources of bias were assessed by examining the methods of randomisation, concealment, blinding, and handling of missing data. Responses for each criterion were scored as yes, no, or unclear. Because the risk of bias can differ across outcomes [25], the scoring was based on pain outcomes whenever possible. For assessor blinding, the scoring was based on studies with performance outcomes and not on outcomes that were self-reported (pain, function and QoL). Funnel plots were used to assess for small study effect/publication bias.

## 3 Results

### 3.1 Characteristics

**Fig. 1:**
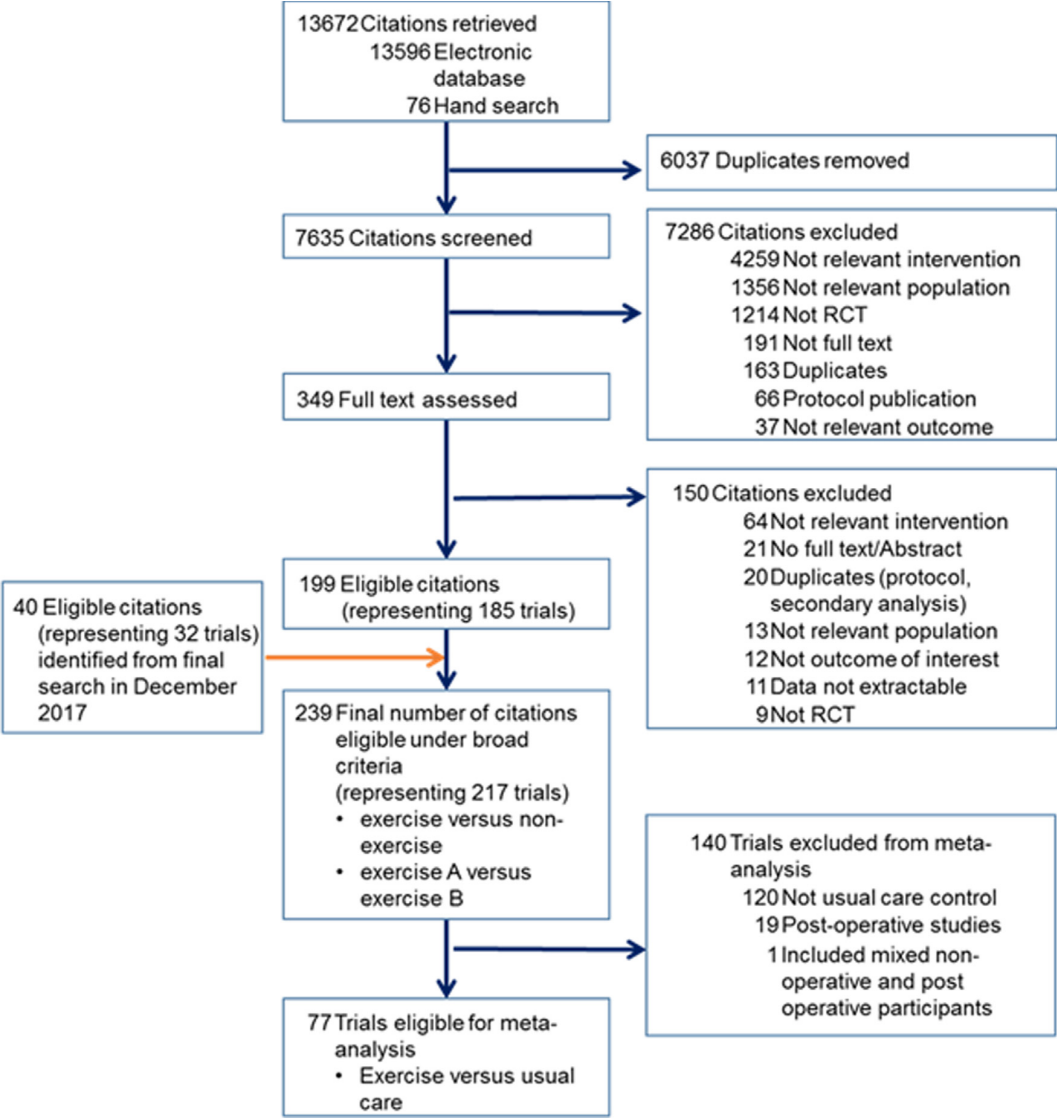
Flow diagram of study selection. RCT, randomised controlled trial.

From the citations retrieved by December 2017, a total of 239 (217 RCTs) met the broad inclusion criteria. However, only 77 RCTs (6472 participants) fulfilled the specific eligibility criteria for the present meta-analysis (see Fig. 1).

The outcome most frequently reported in the trials was objective performance (72 trials; 5913 participants), followed by pain (69 trials; 6072 participants), function (64 trials; 5829 participants), and QoL (34 trials; 3058 participants) (Table 1). The age and sex distributions were similar across all outcomes. Knee OA was the most commonly studied joint site. More than half of the time points reported were < 3 months after start of the intervention, with 8 weeks being the most commonly reported time point. Therefore, we used outcomes at or nearest to 8 weeks after baseline assessment/randomisation as our endpoint for this meta-analysis. The characteristics and risk of bias assessment for each trial are in Appendices 2A and 2B, respecetively.

**Table 1:** Study characteristics of included studies (*n* = 77 trials, 6472 participants).

	Reported outcomes			
	Pain	Function	Performance	QoL
No. of comparisons (no. of RCTs)	70 (69)	65 (64)	73 (72)	34 (34)
No. of participants	6072	5829	5913	3058
% females per trial (range)	73.2 (40.4–100.0)	73.2 (40.4–100.0)	73.3 (40.4–100.0)	72.6 (40.4–100.0)
Age, years, median (range)	64.8 (41.3–84.4)	65 (41.3–84.4)	64.8 (41.3–77.0)	64.9 (49.4–84.4)
BMI, kg/m^2^, median (range)	29.8 (23.8–38.1)	29.8 (23.8–38.1)	29.6 (23.8–38.1)	29.9 (23.8–37.3)
Joint studied (no. of RCTs [%])				
Knee	55 (80)	50 (78)	56 (78)	24 (71)
Hip	8 (12)	9 (14)	9 (13)	7 (21)
Both	6 (9)	5 (8)	7 (10)	3 (9)
OA definition*				
ACR	32	31	35	13
Radiographic	38	37	39	19
Trial data points, months				
1	17	16	16	13
2	26	26	30	9
3	21	17	21	7
6	3	3	1	3
≥ 12	3	3	5	2

QoL: quality of life; BMI: body mass index; ACR: American College of Rheumatology.

* Radiographic diagnosis was mandatory in some studies and some studies merely indicated that ACR criteria was fulfilled.

On preliminary examination of funnel plots, pain and QoL had one outlier each (ES > 5). These studies were excluded from the analyses and are not represented in the funnel plots shown in Appendix 3. Egger's test for publication bias was significant (*P* < 0.05) for all outcomes except QoL.

Because exercise interventions cannot be blinded, the greatest risks of bias were for items related to the blinding of physicians and patients. For 52% of the trials with performance outcomes, blinding of assessors was adequate. Assessor blinding was not scored for RCTs that reported only self-reported outcomes (see Appendix 4). Other items with >50% low risk of bias were related to adequate randomisation (62%), missing outcomes reporting (53%), use of ITT (61%), homogeneity of baseline characteristics (77%), and reporting as pre-specified (95%).

### 3.2 Effect sizes

**Fig. 2:**
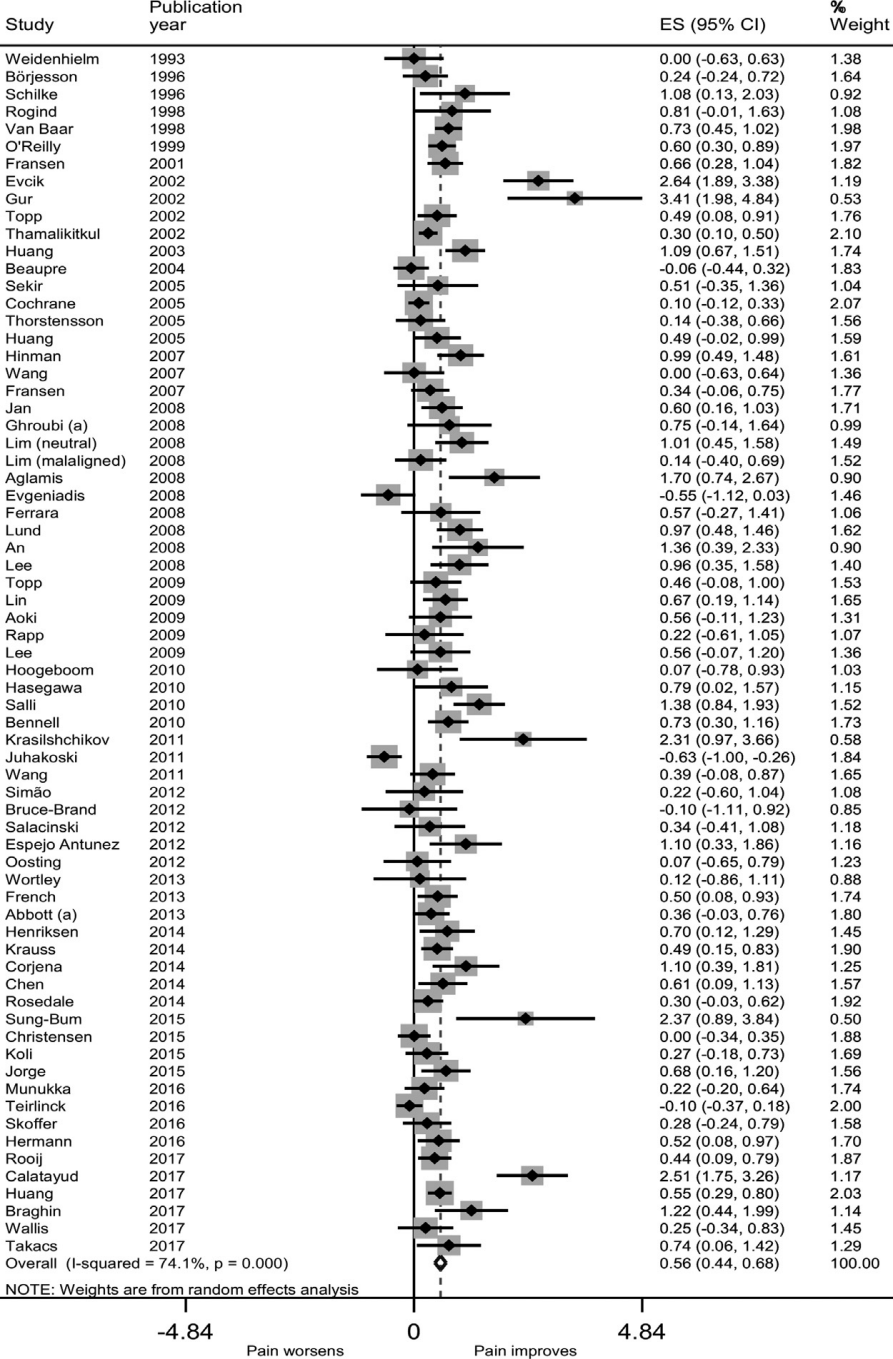
Forest plot of exercise versus usual care for pain.

Relative to usual care at or nearest to 8 weeks, exercise conferred a moderate benefit for pain relief (ES 0.56, 95% CI 0.44–0.68) (Fig. 2), function (0.50, 0.38–0.63), and performance (0.46, 0.35–0.57). A smaller but still statistically significant benefit was observed for QoL (0.21, 0.11–0.31) (Appendix 5).

**Fig. 3:**
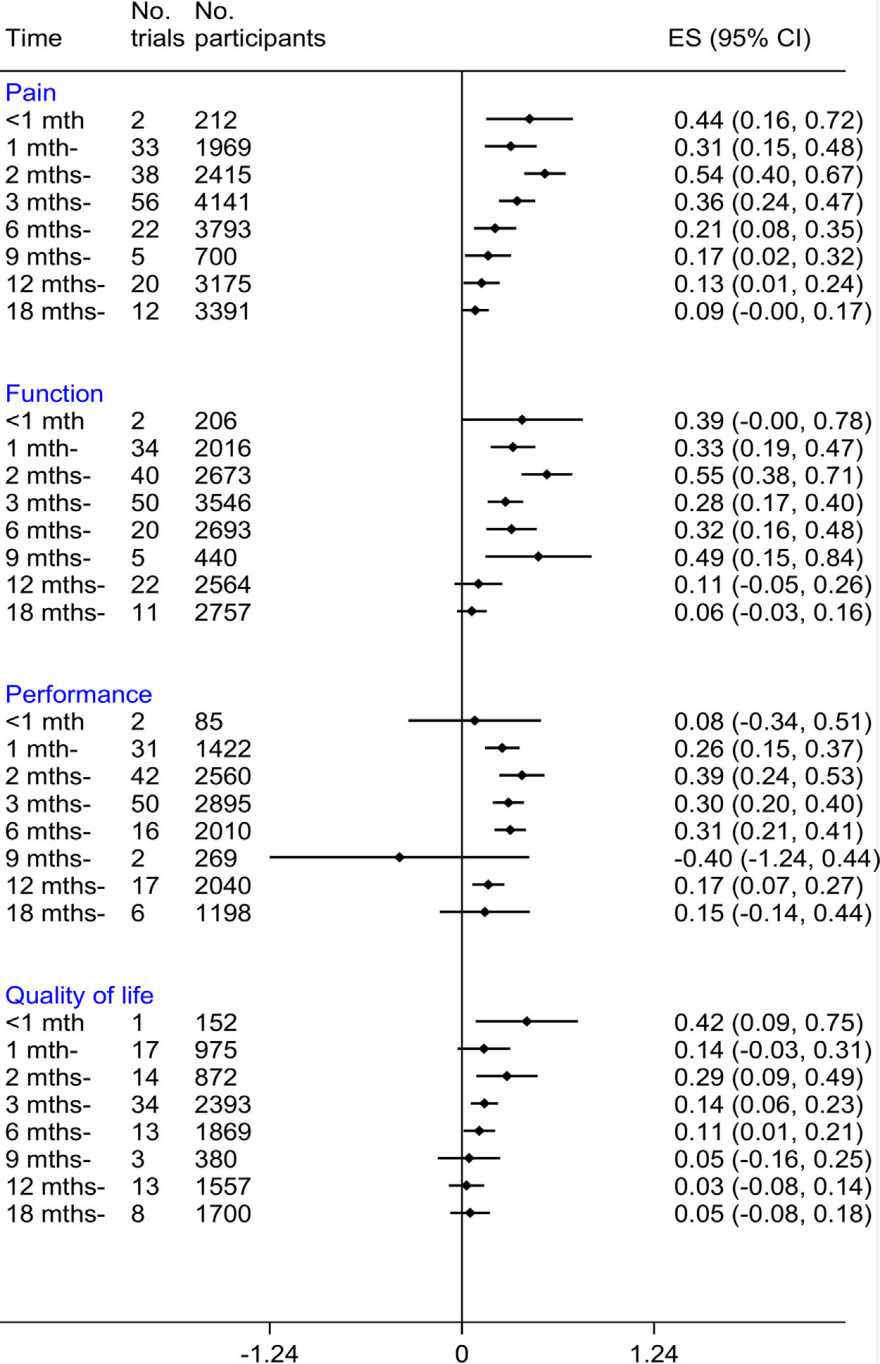
The summary effect of exercise for all outcomes at various times.

We found a general trend for the effects of exercise therapy to peak at 2 months for all outcomes, especially when considering that a reasonable number of trials contributed to the data at each time point (i.e., started from month 1 [Fig. 3]). The effects were reduced gradually after 2 months and became no better than the usual care group at 9 to 18 months depending on the outcome.

### 3.3 Potential determinants

According to the predefined threshold of *P* ≤ 0.10 with the univariate meta-regression in subgroup analysis, we found significantly better pain relief for trials with participants who were younger (< 60 years), with more women, with knee OA and participants not on a waiting list for joint replacement and trials with no/unclear ITT analysis (Table 2). However, after adjustment for covariates on multivariate meta-regression, only younger age, knee OA and not on a waiting list for joint replacement were significant (Table 2).

**Table 2:** Subgroup analysis and potential determinants for pain.

	No. comp. (No. trials)	No. patients	Effect Size (95%CI)	I^2^ (%)	*P*-value	
					Univariate	Multivariate
Overall	69 (68)	5272	0.56 (0.44, 0.68)	74.1		
Age, years						
< 60	12	591	1.32 (0.79–1.86)	86.9	< 0.01*	0.04*
≥ 60	57	4681	0.44 (0.34–0.55)	62.6		
% Female^a^						
<60%	14	1156	0.27 (0.10–0.44)	46.2	0.09*	0.31
≥ 60%	30	2731	0.60 (0.41–0.78)	79.0		
≥ 80%	22	1291	0.56 (0.39–0.74)	50.2		
Mean BMI, kg/m^2^^a^						
< 30	26	2052	0.46 (0.38–0.60)	53.3	0.78	–
≥ 30	18	1187	0.56 (0.28–0.84)	79.8		
Joint						
Knee	55	3750	0.64 (0.51–0.78)	71.2	0.02*	0.10*
Hip	8	703	0.17 (-0.17–0.51)	76.8		
Mixed	6	819	0.43 (0.13–0.72)	73.6		
On TJR waiting list						
No	55	4481	0.62 (0.49–0.75)	74.0	0.10*	0.10*
Yes	14	791	0.33 (0.04–0.63)	73.5		
“Explicit” pain criteria						
None	56	3965	0.59 (0.44–0.73)	76.2	0.64	–
Yes	13	1307	0.49 (0.30–0.68)	62.0		
ACR criteria						
Yes	33	2585	0.64 (0.45–0.82)	78.8	0.39	–
No/unclear	36	2687	0.46 (0.32–0.60)	66.7		
Radiographic requirement						
Yes	39	2649	0.66 (0.46–0.85)	80.9	0.27	–
No/unclear	30	2623	0.46 (0.33–0.59)	53.4		
Adherence monitored						
Yes	45	3961	0.49 (0.36–0.63)	73.2	0.17	–
No/unclear	24	1311	0.73 (0.48–1.00)	74.7		
Analgesic controlled/monitored						
Yes	33	2821	0.55 (0.38–0.73)	77.8	0.86	–
No/unclear	36	2451	0.58 (0.41–0.74)	70.1		
Recruitment centre^a^						
Specialist/hospital	27	1867	0.50 (0.31–0.69)	72.4	0.91	–
GP/community	26	2405	0.50 (0.32–0.67)	72.7		
Mixed	7	680	0.46 (0.30–0.60)	7.1		
ITT use						
Yes	44	3574	0.47 (0.33–0.60)	73.7	0.06*	0.54
No/unclear	25	1698	0.76 (0.55–1.00)	72.7		
> 100/group						
Yes	5	1356	0.31 (0.05–0.58)	82.9	0.25	–
No	64	3916	0.60 (0.46–0.73)	72.5		

95% CI: 95% confidence interval; No. comp.: number of comparators; BMI: body mass index; ACR: American College of Rheumatology; GP: general practitioner; ITT: intent-to-treat; OA: osteoarthritis; TJR: total joint replacement.

* Significant at *P* ≤ 0.10.

a Data were missing in some studies.

For function, response to exercise therapy was better for trials of participants with younger age (mean age < 60 years), knee OA, and without adherence monitoring according to the predefined threshold of *P* ≤ 0.10 in subgroup analysis (Appendix 6A). However, only young age and adherence monitoring remained significant after adjusting for covariates on multivariate meta-regression. A similar process was repeated for performance and QoL outcomes. Apart from trials recruiting from a specific setting/hospital for performance, no other determinants were significant (Appendices 6B and 6C).

### 3.4 Sensitivity analyses

Sensitivity analyses indicated that the effect of exercise across all outcomes was relatively robust. The 95% CIs were overlapping regardless of whether final scores were used instead of change scores, the included studies had imputed SDs, the studies exclusively analysed one index knee per person, or translated publications were included (Appendix 7).

**Fig. 4:**
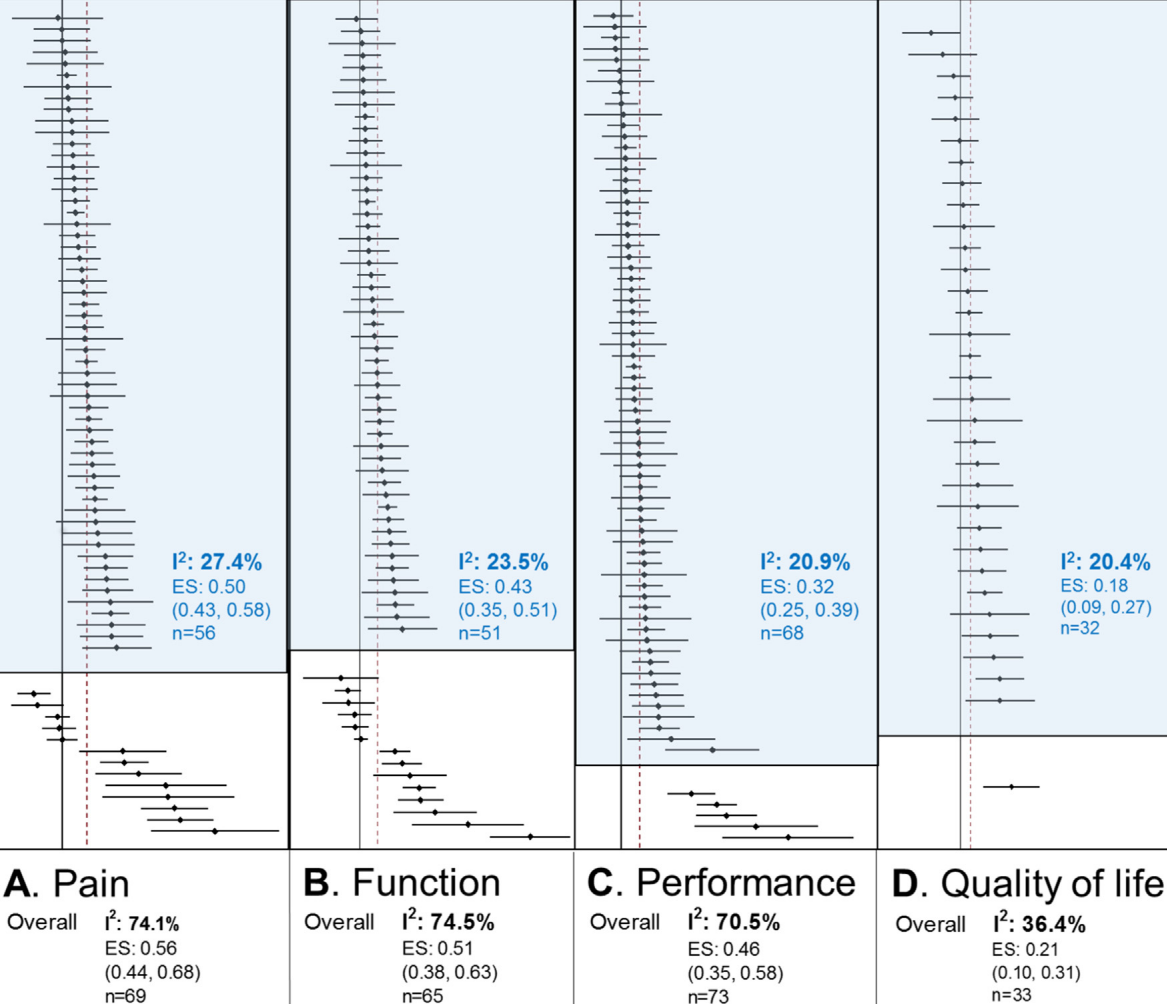
Effect size (ES) for all outcomes by including only “homogeneous” studies (blue shaded area). Blue text: results obtained when sources of heterogeneity (studies in unshaded area) were removed to achieve I^2^ < 30%. Overall results refer to original estimate obtained with all studies. Data in parenthesis represent 95% confidence interval, I^2^: I^2^ statistic, *n*: number of studies.

Additional sensitivity analyses were performed by examining only “homogeneous” RCTs. The 95% CIs for the new pooled ES values obtained with “homogeneous” RCTs still overlapped with those obtained from the primary analysis. With the I^2^ now < 30%, the new ES estimate for pain was 0.50 (95% CI 0.43–0.58), function 0.43 (0.35–0.51), performance 0.32 (0.25–0.39), and QoL 0.18 (0.09–0.27) (Fig. 4). No single factor could be identified to account for the heterogeneity of the outlying RCTs.

## 4 Discussion

This meta-analysis confirmed the findings of previous reports [26], [27], [28] that exercise is superior to usual care for OA at 8 weeks. The benefits of exercise were greater for pain, function, and performance than for QoL. The efficacy of exercise over usual care was generally the greatest at 2 months after starting exercise and were gradually reduced over time to become no better than usual care at 9 to 18 months depending on outcomes. Trials in which the participants were younger, on average; had knee OA; and were not awaiting joint replacement reported better pain relief after exercise therapy. Similarly, trials with younger participants also demonstrated better functional improvement after exercise therapy.

The pattern of the time-dependent effect was similar for all outcomes (i.e., increased in the first 2 months, then gradually reduced thereafter). The greater effect observed within 1 month may be due to the small study effect [29] because only 1 or 2 trials were involved in this estimate. The gradual diminishing efficacy observed after the peak at 2 months agrees with the meta-analysis by Fransen et al. [11]. The authors also examined the longer-term effects of exercise in knee OA and found that the effects on pain and function were reduced by at least half between month 2 and 6 as compared with effects achieved within the first 2 months. One of the reasons for this decline may be poor exercise adherence, in which personal beliefs, social support, relationship with provider and ease of access to exercise facilities play important roles [30].

Overall, the results support the inverse association between exercise benefits and OA severity (i.e., exercise is believed to produce greater improvement with milder than more severe OA) [31]. Patients on waiting lists for surgery, who generally represent OA at the more advanced stage of the clinical spectrum, showed a smaller exercise response as compared with those not on a waiting list. Although we observed smaller exercise benefits in trials with older patients, to what extend this finding is related to OA severity alone is uncertain. In the older population, other age-related conditions (e.g., impaired cellular function, reduced functional reserve in cardiovascular and musculoskeletal systems, and additional health burden of co-morbidities) [32], [33], [34] could also account for the lower effect observed.

For all outcomes, improvement was generally better for participants who were recruited from specialist/hospital centres versus GP/community or mixed recruitment settings. However, recruitment setting was a significant predictor for only performance. Considering that participants recruited from specialist/hospital settings may coincidentally have increased prevalence of severe OA, this finding did not concur with the other analyses supporting greater exercise benefits with milder OA. Instead this result may support the moderating role of exercise facilitators or the context of exercise delivery because such centres would generally be better equipped and perhaps better supported to encourage exercise adherence. However, considering that we found few studies with an unusually large ES (ES >2) within the specialist/hospital subgroup, some caution is warranted in the interpretation.

Similarly, the observation that functional improvement was significantly better in studies without explicit exercise adherence monitoring needs to be interpreted with caution. On one hand, the differentiation may be related to the ease or feasibility of reporting and monitoring of attendance. For example, it may be easier and more reliable to report adherence based on an objective measure of class attendance than self-reported exercise diaries [35]. Also, studies with longer follow-up may be more likely to monitor exercise adherence than studies of short duration. On the other hand, the overall analysis suggests that RCTs with less rigorous methods (small sample size, unclear/no exercise adherence monitoring, no explicit use of ITT) or RCTs that were heterogeneous tended to inflate the effect of exercise.

The effect of exercise on QoL was smaller than that for other outcomes and was not influenced by the type of instrument used (generic or disease specific). One plausible explanation is the difficulty in using a standardised instrument to measure a construct that is individually unique, considering that preferences and expectations vary among individuals [36]. Furthermore, QoL is multidimensional and standardized tools cannot adequately accommodate all factors considered important for all individuals.

Unlike previous meta-analyses, this analysis included a large number of studies, examined more outcomes, and included all types of exercise. To examine effect-modifiers (i.e., determinants), the study focused on exercise programs that were administered alone without additional concurrent treatment. In addition, we limited the control group to usual care and excluded other active non-exercise controls (e.g., patient education or manual therapy) to ensure that the reference arm was standardised. This approach allowed us to explore the effect of potential effect-modifiers (e.g., joint affected, type of exercise, heterogeneous RCTs) by subgroup analysis.

A major limitation of the study is the observational nature of meta-analysis. The results were heavily reliant on the accuracy and comprehensiveness of the primary report. Some issues encountered were missing data on important covariates (such as mean age, sex distribution), and incomplete reporting of group outcomes. Studies with missing data were not included in regression analysis. As a result, the power to perform adjustment for multiple comparisons was limited. Another limitation associated with study-level analyses is ecological bias as well as other problems associated with heterogeneity between studies. Ecological bias occurs because the average change observed at the group level does not accurately reflect the change that occurs within each individual member of the group. Heterogeneity between studies occurs because of variations in patients, disease and methodological characteristics [37]. Particularly for exercise trials, the problem with heterogeneity extends into the interventions used and choice of controls. Despite being widely accepted as a reasonable surrogate for placebo in exercise trials, we have no unified definition of what “usual care” should comprise. With the above limitations, the aim of this analysis was to generate hypotheses to guide future individual patient-data meta-analyses. We had also set a slightly higher statistical significance level (*P* ≤ 0.10) to ensure that we would not miss any potential determinants at this stage [38].

The other limitation is related to the poor quality of clinical trials in exercise. In addition to inadequate blinding of participants and investigators due to the nature of the intervention, a great proportion of exercise RCTs demonstrated high risks of bias in other domains such as reporting bias, allocation concealment and small sample size. The presence of a small study effect in some of the outcomes further downgrades the quality of the evidence. Although a quality assessment tool specifically designed for exercise trials has recently become available [39], no composite scoring system has been developed to grade whether a particular exercise trial is of good or bad quality. Therefore, we could not calculate a single point estimate for exercise effect based on only good-quality studies. Instead, a subset of selected indicators of study quality that we felt were most relevant for exercise trials were used to assess the robustness of our results with subgroup and sensitivity analyses [40].

In conclusion, this meta-analysis compared the efficacy of all types of exercise versus usual care for 4 important knee and hip OA outcomes: pain, function, performance, and QoL. The results showed that, at 8 weeks, exercise had significant moderate benefits for pain and function. Benefits for QoL were small but still significant. The effects often built up to 2 months, then gradually decreased by 9 to 18 months depending on the outcome. Outcomes were better for trials of participants with mean age < 60 years, with knee OA, and not on a waiting list for joint replacement, in a hospital setting, and with no explicit report of adherence monitoring. Further work in individual patient data is still needed to confirm these findings.

## Author contributions

S.L.G. performed search and selection of articles, data extraction, analysis and interpretation and drafted the manuscript. M.S.M.P., J.S. and Y.F. validated data and prepared the manuscript. J.H.L., M.H., M.D. and W.Z. validated study selection and interpreted data. All authors critically reviewed and approved the manuscript. W.Z. conceived project and is the study guarantor.

## Role of funding source

This work was supported by Versus Arthritis (grant numbers 20777, 21595) formerly Arthritis Research UK; University of Malaya (Malaysia); and Ministry of Higher Education (Malaysia).

## Disclosure of interest

The authors declare that they have no competing interest.
