# Supplementary material for: Efficacy and potential determinants of exercise therapy in knee and hip osteoarthritis: A systematic review and meta-analysis
Source: Ann Phys Rehabil Med. 2019 Sep;62(5):356–65. doi: 10.1016/j.rehab.2019.04.006 (PMC6880792; doi:10.1016/j.rehab.2019.04.006)
Supplement: Supplementary file 1 [file mmc1.docx]

**Appendix 1.** MEDLINE search strategy.

1. exercise/ or physical conditioning, human/
2. Circuit-Based Exercise/
3. exercise therapy/ or motion therapy, continuous passive/ or muscle stretching exercises/ or plyometric exercise/ or resistance training/ or hydrotherapy/ or rehabilitation/ or "activities of daily living"/ or dance therapy/
4. muscle strength/ or physical endurance/ or anaerobic threshold/ or exercise tolerance/ or physical fitness/ or postural balance/ or posture/ or psychomotor performance/ or "range of motion, articular"/
5. Pliability/
6. movement/ or motor activity/ or exercise/
7. Physical Exertion/
8. Mind-Body Therapies/
9. running/ or jogging/ or swimming/ or walking/
10. Isometric Contraction/
11. exercise movement techniques/ or breathing exercises/ or qigong/ or tai ji/ or yoga/ or pilates
12. propriocepti$.ab,ti.
13. balanc$.ab,ti.
14. aqua$.ab,ti.
15. cycl$.ab,ti.
16. aerobic.ab,ti.
17. strength$.ab,ti.
18. (tai-ji or taiji or taijiquan or tai ji quan or tai chi or taichi or t ai chi or t'ai chi or tai chi chuan).ab,ti.
19. (qigong or qi gong or chi kung or chikung or ch i kung or ch'i kung).ab,ti.
20. therap$.ab,ti.
21. physiotherap$.ab,ti.
22. train$.ab,ti.
23. neuromuscular training.ab,ti.
24. treadmill.ab,ti.
25. 1 or 2 or 3 or 4 or 5 or 6 or 7 or 8 or 9 or 10 or 11 or 12 or 13 or 14 or 15 or 16 or 17 or 18 or 19 or 20 or 21 or 22 or 23 or 24
26. Osteoarthritis, Hip/ or Osteoarthritis, Knee/
27. coxarthritis.mp.
28. coxarthr$.ab,ti.
29. gonarthr$.ab,ti.
30. (knee$ adj3 pain).ab,ti.
31. (hip adj3 pain).ab,ti.
32. osteoarthr$.ab,ti.
33. degenerative joint disease?.ab,ti.
34. osteoarthritis/
35. (Osteoarthriti$ or OA or osteo arthriti$ or osteoarthros$ or osteo arthros$ or arthropath$ or arthrosis or arthroses).ti,ab.
36. Hip Joint/
37. Hip/
38. hip$.ab,ti.
39. Knee/
40. knee$.ab,ti.
41. knee joint/ or patellofemoral joint/
42. 26 or 27 or 28 or 29 or 30 or 31
43. 32 or 33 or 34 or 35
44. 36 or 37 or 38 or 39 or 40 or 41
45. 43 and 44
46. 42 or 45
47. randomized controlled trial.pt.
48. controlled clinical trial.pt.
49. (placebo or (standard adj3 care)).ab.
50. clinical trials as topic.sh.
51. random$.ab,ti.
52. trial$.ab,ti.
53. RCT.ab,ti.
54. 47 or 48 or 49 or 50 or 51 or 52 or 53
55. limit 54 to humans
56. 25 and 46 and 55

**Appendix 2A.** Characteristics of included studies and references.

| Study | Age | BMI | N (fem %) | Jt | Diagn. | Pre-surg. | Study arms | | |  | Tool and outcome time point, month | | | |  |
| --- | --- | --- | --- | --- | --- | --- | --- | --- | --- | --- | --- | --- | --- | --- | --- |
|  |  |  |  |  |  |  | **1** | **2** | **3** |  | **Pain** | **Function** | **Performance** | **QoL** | |
| ^1^Abbott 2013 | 67 (10) | 29 (6) | 206 (55) | K,H | A | N | MEx | UC | - |  | Pain intensity score (0-10), 12 | WOMAC (0-240), 2.25 | 40m walk time (s), 12 | - | |
| ^2^Aglamis 2008^†^ *^3^Aglamis 2009* | 56 (5) | 33 (5) | 31 (100) | K | A, I | N | MEx | UC | - |  | VAS (0-10), 1.5 | WOMAC (total), 1.5 | 6MWT (m), 1.5 | SF-36 (0-100) General health (0-100), 1.5 | |
| ^4^An 2008 | 65 (7) | 26 (3) | 28 (100) | K | A | N | MB | UC | - |  | WOMAC pain, 2 | WOMAC (physical fx), 2 | 6MWT (m), 2 | SF-36 Mental health (0-100), 2 | |
| ^5^Aoki 2009 | 73 (6) | 26 (3) | 36 (100) | K | I | Y | Fl/Sk | UC | - |  | VAS (0-100), 3 | - | Walking speed (m/min), 3 | - | |
| ^6^Arnold 2010 | 74 (6) | 30 (5) | 83 (68) | H | A | N | MEx | UC | - |  | Arthritis Impact measurement scale -2, results not reported | Activity specific balance confidence (0-100), 2.75 | 6MWT (m), 2.75 | - | |
| ^7^Beaupre 2004 | 67 (7) | 32 (6) | 131 (55) | K | UClr | Y | Str | UC | - |  | WOMAC pain (0-100), 1.5 | WOMAC fx (0-100), 1.5 | Quadriceps strength, 1.5 | SF-36 Mental health, 1.5 | |
| ^8^Bennell 2010 | 65 (8) | 28 (4) | 89 (48) | K | A, I | N | Str | UC | - |  | NRS (0-10), 3.25 | WOMAC fx (0-68), 3.25 | Timed stair task (s), 3.25 | - | |
| ^9^Börjesson 1996 | 64 (5) | - (-) | 68 (50) | K | I | Y | MEx | UC | - |  | NRS (0-10), 3 | - | Walking speed (m/s), 3 | - | |
| ^10^Braghin 2017 | 60 (-) | 31 (-) | 42 (74) | K | I | N | MEx | UC | - |  | WOMAC pain, 2 | WOMAC fx, 2 | Timed movement (s), 2 | - | |
| ^11^Bruce-Brand 2012 | 64 (5) | 33 (4) | 26 (42) | K | I | Y | Str | UC | - |  | WOMAC pain (0-20), 1.5 | WOMAC fx, 1.5 | Timed walk (s), 1.5 | SF-36 MCS, 1.5 | |
| ^12^Calatayud 2017 | 50 (-) | 32 (-) | 50 (-) | K | A, I | Y | Str | UC | - |  | VAS (0-10), 2 | WOMAC fx, 2 | Timed up and go (s), 2 | SF-36 Physical fx, 2 | |
| ^13^Chen 2014 | 63 (7) | - (-) | 120 (85) | K | A, I | N | Str | UC | - |  | VAS (0-10), 2 | Lequesne's index (1-26), 2 | Knee range of motion, 2 | - | |
| ^14^Chopp-Hurley 2017 | 54 (-) | 29 (-) | 24 (79) | K,H | A | N | Str | UC | - |  | ICOAP, not extractable | HOOS/KOOS, not extractable | 6MWT (m), 3 | HOOS/KOOS, not extractable | |
| ^15^Christensen 2015^†^ *^16^Henriksen 2014* | 63 (6) | 37 (-) | 192 (81) | K | I | N | MEx | UC | - |  | VAS (0-100), 12 | KOOS (daily fx) (0-100), 12 | 6MWT (m), 12 | SF-36 Mental health (0-100), 12 | |
| ^17^Cochrane 2005 | 70 (7) | 30 (-) | 312 (63) | K,H | UClr | N | MEx | UC | - |  | WOMAC pain (0-96), 6 | WOMAC fx (0-96), 6 | Timed walk (s), 12 | SF-36 Mental health (0-100), 6 | |
| ^18^Cheung 2014 | 72 (6) | 29 (-) | 36 (100) | K | A | N | MB | UC | - |  | WOMAC pain (0-20), 2 | WOMAC fx (0-68), 2 | 8 min walk (m), 2 | SF12 MCS (0-100), 2 | |
| ^19^D'Lima 1996 | 70 (6) | - (-) | 30 (47) | K | UClr | Y | Ae | Mex | UC |  | - | Hospital for Special Surgery Knee rating (0-100), 1.5 | - | - | |
| ^20^Espejo Antunez 2012 | 84 (8) | - (-) | 31 (77) | K | A | N | MEx | UC | - |  | VAS, 1 | WOMAC fx (0-96), 1 | - | SF-36 Mental health, 1 | |
| ^21^Evcik 2002 | 56 (6) | - (-) | 90 (62) | K | I | N | Ae | UC | - |  | VAS (0-100), 6 | WOMAC fx (0-68), 6 | - | NHP - emotional reaction, 6, Outlier | |
| ^22^Evgeniadis 2008 | 68 (4) | 34 (-) | 53 (79) | K | A, I | Y | Str | UC | - |  | SF-36 body pain, 1 | SF-36 physical fx, 1 | Active range of motion (flexion), 1 | SF-36 Mental health, 1 | |
| ^23^Ferrara 2008 | 63 (8) | - (-) | 23 (61) | H | UClr | Y | MEx | UC | - |  | VAS (0-10), 1 | WOMAC fx (0-68), 1 | Quads strength, 1 | SF-36 Mental health, 1 | |
| ^24^Fransen 2001 | 67 (9) | 29 (5) | 126 (73) | K | I | N | MEx | UC | - |  | WOMAC pain (0-100), 2 | WOMAC fx (0-100), 2 | Gait speed (cm/s), 2 | SF-36 Mental health, 2 | |
| ^25^Fransen 2007 | 70 (6) | 30 (-) | 152 (74) | K,H | A | N | MB | MEx | UC |  | WOMAC pain (0-100), 3 | WOMAC fx (0-100), 3 | Timed walked (s), 3 | SF-12 MCS (Mean+/- SD 50+/-10), 3 | |
| ^26^French 2013 | 62 (10) | - (-) | 131 (64) | H | A, I | N | MEx | UC | - |  | NRS, 2 | WOMAC fx (0-68), 2 | 50 ft walk test (s), 2 | SF-36 Mental health (0-100), 2 | |
| ^27^Ghroubi 2008 (a) | 41 (10) | 38 (-) | 56 (-) | K | I | N | MEx | UC | - |  | VAS pain(0-10), 2 | WOMAC, 2 | 6MWT (m), 2 | - | |
| ^28^Gur 2002 | 56 (12) | - (-) | 23 (-) | K | I | N | Str | UC | - |  | NRS (0-10), 2 | NRS of functional capacity (0-10), 2 | 15m walk (s), 2 | - | |
| ^29^Hasegawa 2010 | 77 (4) | 24 (4) | 28 (64) | K | UClr | N | MEx | UC | - |  | NRS (0-10), 3 | - | Time up and go (s), 3 | - | |
| ^30^Henriksen 2014a^† 31^*Henriksen 2017* | 64 (8) | 29 (-) | 60 (80) | K | I | N | MEx | UC | - |  | KOOS pain (0-100), 3 | KOOS fx (0-100), 3 | Walking speed (m/s), 3 | KOOS QOL (0-100), 3 | |
| ^32^Hermann 2016 | 70 (8) | 28 (5) | 80 (65) | H | UClr | Y | Str | UC | - |  | HOOS pain, 2.5 | HOOS ADL, 2.5 | Quadriceps power, 2.5 | HOOS QoL, 2.5 | |
| ^33^Hinman 2007 | 62 (9) | 33 (7) | 71 (68) | K,H | A, I | N | MEx | UC | - |  | VAS (0-10) on movement, 1.5 | WOMAC fx (0-1700), 1.5 | 6MWT (m), 1.5 | Assessment of QoL (-0.04 to1), 1.5 | |
| ^34^Hoogeboom 2010 | 76 (4) | 27 (-) | 21 (67) | H | UClr | Y | MEx | UC | - |  | VAS, 1 | HOOS ADL fx (0-100), 1 | 6MWT (m), 1 | HOOS QoL (0-100), 1 | |
| ^35^Huang 2003 | 62 (5) | - (-) | 132 (71) | K | A, I | N | MEx | UC | - |  | VAS (0-10), 2 | Lequesne index, 2 | Walking speed (m/s), 2 | - | |
| ^36^Huang 2005 | 65 (6) | - (-) | 140 (81) | K | A | N | Str | UC | - |  | VAS (standing/walking) (0-10), 2 | Lequesne index (1-26), 2 | Walking speed (m/min), 2 | - | |
| ^37^Huang 2017 | 68 (6) | 25 (2) | 250 (80) | K | A | N | Str | UC | - |  | VAS (0-100), 3 | WOMAC (0-68), 3 | - | - | |
| ^38^Hunt 2013 | 66 (11) | 27 (-) | 17 (53) | K | A, I | N | Str | UC | - |  | - | - | Walking speed (m/s), 2.5 | - | |
| ^39^Jan 2008 | 63 (7) | - (-) | 98 (81) | K | A, I | N | Str | UC | - |  | WOMAC pain (0-20), 2 | WOMAC fx (0-68), 2 | Timed walk (s), 2 | - | |
| ^40^Jan 2009 | 63 (7) | - (-) | 106 (69) | K | A, I | N | Str | UC | - |  | - | WOMAC fx (0-68), 2 | Timed walk (s), 2 | - | |
| ^41^Jorge 2015 | 61 (7) | 31 (4) | 60 (100) | K | A | N | Str | UC | - |  | VAS (0-10), 1.5 | WOMAC (0-68), 1.5 | 6MWT (m), 1.5 | SF-36 Mental health (0-100), 1.5 | |
| ^42^Juhakoski 2011 | 67 (6) | - (-) | 118 (70) | H | A, I | N | MEx | UC | - |  | WOMAC pain (0-100), 3 | WOMAC fx (0-100), 3 | 6MWT (m), 3 | SF-36, not reported | |
| ^43^Koli 2015 | 59 (4) | 27 (4) | 78 (100) | K | I | N | Ae | UC | - |  | KOOS pain (0-100), 12 | KOOS fx (0-100), 12 | Strength, 12 | KOOS QOL (0-100), 12 | |
| ^44^Krasilshchikov 2011 | 58 (5) | 28 (5) | 16 (100) | K | I | N | MEx | UC | - |  | WOMAC pain, 2 | WOMAC fx, 2 | 6MWT (m), 2 | - | |
| ^45^Krauss 2014^† 46^*Steinhilber 2017* | 59 (10) | 27 (4) | 218 (40) | H | A | N | MEx | UC | - |  | WOMAC pain (0-100), 3 | WOMAC fx (0-100), 3 | Hip abd peak torque, 3 | SF-36 Mental health (0-100), 3 | |
| ^47^Kreindler 1989 | - (-) | - (-) | 32 (75) | K | UClr | N | Str | UC | - |  | - | - | Quadriceps strength (180), 1.5 | - | |
| ^48^Lee 2008 | 76 (6) | - (-) | 46 (78) | K | UClr | N | MB | UC | - |  | WOMAC pain (0-96), 3 | WOMAC fx (0-96), 3 | Timed up and go test (s), 3 | - | |
| ^49^Lee 2009 | 69 (5) | - (-) | 44 (93) | K | I | N | MB | UC | - |  | WOMAC pain (26-130), 2 | WOMAC fx (0-100), 2 | 6MWT (m), 2 | SF-36 Mental health (0-100), 2 | |
| ^50^Lim 2008* (neutral, malaligned) | 66 (8) | 29 (-) | 107 (55) | K | A, I | N | Str | UC | - |  | WOMAC pain (0-100), 3 | WOMAC fx (0-100), 3 | Stair climb (s), 3 | - | |
| ^51^Lin 2009 | 63 (7) | - (-) | 108 (69) | K | I | N | Fl/Sk | Str | UC |  | WOMAC pain (0-20), 2 | WOMAC fx (0-68), 2 | Timed walk (s), 2 | - | |
| ^52^Lund 2008 | 68 (14) | - (-) | 79 (79) | K | A | N | MEx | UC | - |  | Vas at rest (0-100), 2 | KOOS ADL (0-100), 2 | Strength, 2 | - | |
| ^53^Moghadam 2017 | 67 (-) | 25 (-) | 20 (-) | K | A | N | Ae | UC | - |  | - | - | 6MWT (m), 2 | - | |
| ^54^Munukka 2016^†^  ^55^*Waller 2017* | 64 (-) | 27 (-) | 87 (100) | K | I | N | Str | UC | - |  | KOOS pain (0-100), 4 | KOOS ADL (0-100), 4 | Walking speed (m/s), 4 | KOOS QoL (0-100), 4 | |
| ^56^Oida 2008 | 74 (5) | 25 (4) | 88 (86) | K | I | N | MEx | UC | - |  | - | WOMAC (0-300), 3 | Timed walk (s), 3 | - | |
| ^57^Oosting 2012 | 76 (6) | 28 (-) | 30 (80) | H | UClr | Y | MEx | UC | - |  | NRS (0-10), 1.25 | HOOS ADL fx (0-100), 1.25 | 6 MWT (m), 1.25 | HOOS QoL (0-100), 1.25 | |
| ^58^O'Reilly 1999 | 62 (10) | - (-) | 180 (66) | K | UClr | N | Str | UC | - |  | WOMAC pain (0-20), 6 | WOMAC fx (0-68), 6 | Quadriceps strength, 6 | SF-36 Mental health, 6 | |
| ^59^Rapp 2009 | 60 (8) | - (-) | 39 (56) | K | A | N | Str | UC | - |  | VAS (0-10), 2 | - | Strength, 2 | - | |
| ^60^Rogind 1998 | 71 (7) | 27 (4) | 25 (84) | K | A, I | N | MEx | UC | - |  | VAS at rest (0-10), 3 | Algofunctional index, 3 | 20m walk speed (m/s), 3 | - | |
| ^61^ Rooij 2016 | 64 (-) | 36 (-) | 126 (75) | K | A | N | MEx | UC | - |  | NRS (0-10), 2.5 | WOMAC fx (0-68), 2.5 | 6MWT (m), 2.5 | - | |
| ^62^Rosedale 2014 | 65 (10) | 31 (7) | 158 (56) | K | I | Y | MEx | UC | - |  | KOOS pain (0-100), 3 | KOOS fx (0-100), 3 | - | KOOS QoL (0-100), 3 | |
| ^63^Salacinski 2012 | 58 (10) | 24 (-) | 37 (73) | K | I | N | Ae | UC | - |  | VAS rest (0-100), 3 | WOMAC fx (0-100), 3 | Walking speed (m/s), 3 | KOOS-QoL (0-100), 3 | |
| ^64^Salli 2010 ^65^*Salli 2006* | 57 (7) | 32 (-) | 75 (77) | K | A, I | N | Str | UC | - |  | VAS (rest), 2 | WOMAC fx, 2 | Strength (180^o^/s peak torque), 2 | SF-36 Mental health, 2 | |
| ^66^Schilke 1996 | 66 (-) | - (-) | 23 (74) | K | UClr | N | Str | UC | - |  | Osteoarthritis screening index pain, 2 | Arthritis Impact Measurement Scales activity, 2 | Strength, 2 | - | |
| ^67^Sekir 2005 | 60 (9) | - (-) | 22 (73) | K | A | N | Fl/Sk | UC | - |  | VAS (0-100) after inactivity, 1.5 | NRS subjective functional rating (0-10), 1.5 | Timed walk (s), 1.5 | - | |
| ^68^Simão 2012 | 72 (6) | 28 (-) | 35 (80) | K | A, I | N | Str | UC | - |  | WOMAC pain (0-500), 3 | WOMAC fx (0-1700), 3 | 6MWT (m), 3 | - | |
| ^69^Skoffer 2016 | 70 (-) | 31 (-) | 59 (61) | K | I | Y | Str | UC | - |  | KOOS pain (0-100), 1 | KOOS ADL (0-100), 1 | 6MWT (m), 1 | KOOS QoL (0-100), 1 | |
| ^70^Sung-Bum 2015 | 65 (3) | - (-) | 14 (100) | K | A, I | N | Fl/Sk | UC | - |  | VAS, 2-mth | - | Strength (isokinetic), 2 | - | |
| ^71^Swank 2011 | 63 (7) | 34 (-) | 71 (65) | K | UClr | Y | MEx | UC | - |  | VAS (after walk test) (1-10), 1.5, outlier | - | 6MWT (m), 1.5 | - | |
| ^72^Takacs 2017 | 67 (-) | 29 (-) | 40 (80) | K | I | N | Fl/Sk | UC | - |  | NRS (0-10), 2.5 | WOMAC fx (0-68), 2.5 | Composite peak lower limb strength, 2.5 | - | |
| ^73^Teirlinck 2016 | 66 (-) | 28 (-) | 203 (59) | H | A | N | MEx | UC | - |  | NRS (0-10), 1.5 | HOOS fx (0-100), 1.5 | Timed up and go (s), 12 | EQ-5D, 1.5 | |
| ^74^Kuptniratsaikul 2002 | 68 (6) | - (-) | 392 (78) | K | I | N | Str | UC | - |  | Pain score, 2 | Functional incapacity score (0-20), 2 | 6MWT (m), 2 | - | |
| ^75^Thorstensson 2005 | 56 (6) | 30 (-) | 61 (51) | K | I | N | MEx | UC | - |  | KOOS pain (0-100), 1.5 | KOOS (ADL) (0-100), 1.5 | One leg semi squatting, 1.5 | SF-36 Mental health, 1.5 | |
| ^76^Topp 2002 | 63 (11) | - (-) | 102 (73) | K | A | N | Str | UC | - |  | WOMAC pain (0-20), 4 | WOMAC fx (0-68), 4 | Upstairs (s), 4 | - | |
| ^77^Topp 2009 | 64 (7) | 32 (6) | 54 (69) | K | UClr | Y | MEx | UC | - |  | VAS (0-10) at 6 MWT, 1 | - | 6MWT (m), 1 | - | |
| ^78^Van Baar 1998^† 79^*Van Baar 2001* | 68 (9) | - (-) | 201 (78) | K,H | A | N | MEx | UC | - |  | VAS past week 0-100), 3 | Influence of Rheumatic disease on general health and lifestyle disability (-28--7), insufficient data | Muscle strength (knee), 3 | - | |
| ^80^Wallis 2017 | 68 (-) | 34 (-) | 46 (44) | K | I | N | Ae | UC | - |  | NRS (0-10), 3 | WOMAC ADL(0-68), 3 | 40m walk test (m/s), 3 | EQ5D (0-1), 3 | |
| ^81^Wang 2007 | 66 (12) | - (-) | 38 (84) | K,H | UClr | N | MEx | UC | - |  | VAS (0-100), 1.5 | Multidimensional health assessment questionnaire, 1.5 | Strength knee extension, 1.5 | - | |
| ^82^Wang 2011 | 68 (6) | 26 (2) | 78 (86) | K | UClr | N | MEx | UC | - |  | KOOS pain (0-100), 1.5 | KOOS ADL (0-100), 1.5 | 6MWT (m), 1.5 | KOOS QOL (0-100), 1.5 | |
| ^83^Weidenhielm 1993 | 64 (5) | - (-) | 39 (51) | K | UClr | Y | MEx | UC | - |  | Pain 10-grade scale (walking), 3 | - | Max Walking speed (m/min), 3 | - | |
| ^84^Wortley 2013 | 69 (6) | 32 (6) | 31 (71) | K | A, I | N | MB | Str | UC |  | WOMAC pain, 2.5 | WOMAC fx, 2.5 | 6 MWT (m), 2.5 | - | |

^*^ Two sets of comparison were obtained

^†^ Trials with multiple reporting available

ADL, activity of daily living; BMI, body mass index; fem, female; Jt, joint; QoL, quality of life; Ucl, unclear; K, knee; H, hip; A,American College of Rheumatology criteria; I, Imaging criteria; Y, Yes; N, No; Ae, aerobic; Fl/Sk, flexibility and skills; MB, mind-body; MEx, mixed exercise; Str, strength; VAS, visual analogue scale; WOMAC, Western Ontario & McMaster Universities Osteoarthritic Index; 6MWT, 6-min walk test; fx, function; NRS, numeric rating score; KOOS, Knee injury and Osteoarthritis Outcome Score; HOOS, Hip disability and Osteoarthritis Outcome Score

**Appendix 2B.** Risk of bias assessment per article and country of publication.

| **Study** | **Adequate**  **randomization** | **>100 per group** | **Concealed**  **Allocation** | **Physician**  **Blind** | **Patient**  **Blind** | **Assessor**  **Blind** | **Missing**  **Outcome**  **Assess** | **Intention-**  **to-treat**  **Use** | **Homogenous**  **Group** | **Reported**  **as Pre-**  **specified** | **Country** |
| --- | --- | --- | --- | --- | --- | --- | --- | --- | --- | --- | --- |
| 1. ^1^Abbott 2013 | Yes | No | Unclear | No | No | Yes | Yes | Yes | Yes | Yes | New Zealand |
| 1. ^2^Aglamis 2008 | Yes | No | Unclear | Unclear | No | Yes | No | No | Unclear | Yes | Turkey |
| 1. ^4^An 2008 | Unclear | No | Unclear | Unclear | No | No | Yes | No | Yes | Yes | China |
| 1. ^5^Aoki 2009 | Unclear | No | Unclear | Unclear | No | Yes | Yes | Yes | Yes | Yes | Japan |
| 1. ^6^Arnold 2010 | Yes | No | Yes | No | No | Yes | Yes | Yes | Yes | Yes | Canada |
| 1. ^7^Beaupre 2004 | Unclear | No | Yes | No | No | Yes | Yes | Yes | Yes | Yes | Canada |
| 1. ^8^Bennell 2010 | Yes | No | Yes | No | No | Yes | Yes | Yes | Yes | Yes | Australia |
| 1. ^9^Börjesson 1996 | Yes | No | Unclear | No | No | Unclear | Yes | Yes | Yes | Unclear | Sweden |
| 1. ^10^Braghin 2017 | Yes | No | Yes | No | No | Unclear | Unclear | Unclear | Unclear | Yes | Brazil |
| 1. ^11^Bruce-Brand 2012 | Yes | No | Unclear | No | No | Yes | Unclear | Unclear | Yes | No | United Kingdom |
| 1. ^12^Calatayud 2017 | Yes | No | Unclear | No | No | Unclear | Yes | Yes | Yes | Yes | Denmark |
| 1. ^13^Chen 2014 | Yes | No | Yes | No | No | Unclear | Unclear | Yes | Unclear | Yes | Taiwan |
| 1. ^14^Chopp-Hurley 2017 | Yes | No | Unclear | No | No | Yes | No | Unclear | Yes | Yes | Canada |
| 1. ^15^Christensen 2015 | Yes | No | Yes | No | No | Yes | Yes | Yes | Yes | Yes | Denmark |
| 1. ^17^Cochrane 2005 | Yes | Yes | Yes | No | No | Yes | Yes | Yes | Yes | Yes | United Kingdom |
| 1. ^18^Cheung 2014 | Yes | No | Unclear | No | No | Yes | Yes | Yes | Yes | Yes | United States |
| 1. ^19^D'Lima 1996 | Yes | No | Unclear | Unclear | No | Unclear | Yes | Unclear | No | Yes | United States |
| 1. ^20^Espejo Antunez 2012 | Yes | No | No | No | No | Yes | Yes | Yes | Unclear | Yes | Spain |
| 1. ^21^Evcik 2002 | Unclear | No | Unclear | No | No | No | No | No | Yes | Yes | Turkey |
| 1. ^22^Evgeniadis 2008 | Yes | No | Yes | No | No | Unclear | No | Yes | Yes | Yes | Greece |
| 1. ^23^Ferrara 2008 | Yes | No | Unclear | Unclear | No | Yes | Unclear | Yes | Yes | No | Italy |
| 1. ^24^Fransen 2001 | Yes | No | Yes | No | No | Yes | Yes | Yes | Yes | Yes | Australia |
| 1. ^25^Fransen 2007 | Yes | No | Unclear | No | No | Yes | Yes | Yes | Yes | Yes | Australia |
| 1. ^26^French 2013 | Yes | No | Yes | No | No | Yes | Yes | Yes | Unclear | Yes | Ireland |
| 1. ^27^Ghroubi 2008 (a) | Unclear | No | Unclear | No | No | Unclear | No | No | Yes | Yes | Tunisia |
| 1. ^28^Gur 2002 | Unclear | No | Unclear | No | No | Unclear | Yes | Yes | Unclear | Yes | Turkey |
| 1. ^29^Hasegawa 2010 | Unclear | No | Unclear | No | No | Unclear | Yes | Yes | Yes | Yes | Japan |
| 1. ^30^Henriksen 2014 | Yes | No | Yes | No | No | Yes | No | No | No | Yes | Denmark |
| 1. ^32^Hermann 2016 | Yes | No | Yes | No | No | Unclear | Yes | Yes | Yes | Yes | Denmark |
| 1. ^33^Hinman 2007 | Yes | No | Yes | No | No | Yes | Yes | Yes | Yes | Yes | Australia |
| 1. ^34^Hoogeboom 2010 | Unclear | No | Yes | No | No | Yes | Yes | Yes | Yes | Yes | Netherland |
| 1. ^35^Huang 2003 | Unclear | No | Yes | No | No | Unclear | No | No | Unclear | Yes | Taiwan |
| 1. ^36^Huang 2005 | Unclear | No | Yes | No | No | Unclear | No | No | Unclear | Yes | Taiwan |
| 1. ^37^Huang 2017 | Yes | Yes | No | Unclear | No | No | Unclear | Unclear | Yes | Yes | China |
| 1. ^38^Hunt 2013 | Yes | No | Yes | No | No | Yes | No | Unclear | Yes | Yes | Canada |
| 1. ^39^Jan 2008 | Yes | No | Unclear | No | No | Yes | No | Yes | Yes | Yes | Taiwan |
| 1. ^40^Jan 2009 | Yes | No | Unclear | No | No | Yes | Unclear | Yes | Yes | Yes | Taiwan |
| 1. ^41^Jorge 2015 | Yes | No | Yes | No | No | Yes | Yes | Yes | Yes | Yes | Brazil |
| 1. ^42^Juhakoski 2011 | Yes | No | Yes | No | No | Yes | Yes | Yes | Yes | Yes | Finland |
| 1. ^43^Koli 2015 | Yes | No | Unclear | No | No | Yes | No | No | Yes | Yes | Finland |
| 1. ^44^Krasilshchikov 2011 | Unclear | No | Unclear | No | No | Yes | Unclear | Unclear | Yes | Yes | Malaysia |
| 1. ^45^Krauss 2014 | Unclear | No | Yes | No | No | No | Yes | Yes | Yes | Yes | Germany |
| 1. ^47^Kreindler 1989 | No | No | No | No | No | Unclear | Yes | Yes | Yes | Yes | United States |
| 1. ^48^Lee 2008 | Unclear | No | Unclear | No | No | Unclear | No | No | No | Yes | Korea |
| 1. ^49^Lee 2009 | Yes | No | Yes | No | No | Yes | Yes | Yes | Yes | Yes | Korea |
| 1. ^50^Lim 2008 (neutral) | Yes | No | Unclear | No | No | Yes | Yes | Yes | Yes | Yes | Australia |
| 1. ^51^Lin 2009 | Yes | No | Yes | No | No | Yes | Yes | Yes | Yes | Yes | Taiwan |
| 1. ^52^Lund 2008 | Unclear | No | Yes | No | No | Yes | Yes | Yes | Yes | Yes | Denmark |
| 1. ^53^Moghadam 2017 | Unclear | No | Unclear | No | No | Unclear | No | Unclear | Yes | Unclear | Azerbaijan |
| 1. ^54^Munukka 2016 | Yes | No | Unclear | No | No | Yes | Yes | Yes | Yes | Yes | Finland |
| 1. ^56^Oida 2008 | Yes | No | Unclear | No | No | Unclear | Unclear | No | No | Yes | Japan |
| 1. ^57^Oosting 2012 | Unclear | No | Yes | No | No | Yes | No | Yes | Yes | Yes | Netherlands |
| 1. ^58^O'Reilly 1999 | Yes | No | Yes | No | No | Unclear | Unclear | Yes | Yes | Yes | United Kingdom |
| 1. ^59^Rapp 2009 | Unclear | No | Unclear | No | No | Unclear | Yes | Yes | Unclear | Yes | Germany |
| 1. ^60^Rogind 1998 | Yes | No | Unclear | No | No | Yes | Unclear | Yes | Yes | Unclear | Denmark |
| 1. ^61^Rooij 2016 | Yes | No | Yes | No | No | Yes | Yes | Yes | Yes | Yes | Netherlands |
| 1. ^62^Rosedale 2014 | Yes | No | Yes | No | No | No | Yes | Yes | Yes | Yes | Canada |
| 1. ^63^Salacinski 2012 | Yes | No | Unclear | No | No | Unclear | No | No | No | Yes | United States |
| 1. ^64^Salli 2010 | Unclear | No | Yes | No | No | Unclear | No | No | Yes | Yes | Turkey |
| 1. ^66^Schilke 1996 | Unclear | No | Unclear | No | No | Unclear | No | No | Unclear | Yes | United States |
| 1. ^67^Sekir 2005 | Unclear | No | Unclear | No | No | Unclear | Yes | Yes | Unclear | Yes | Turkey |
| 1. ^68^Simão 2012 | Unclear | No | Yes | No | No | Yes | No | Yes | No | Yes | Brazil |
| 1. ^69^Skoffer 2016 | Yes | No | Yes | No | No | Yes | Yes | Yes | Yes | Yes | Denmark |
| 1. ^70^Sung-Bum 2015 | Unclear | No | Unclear | No | No | Unclear | Unclear | Unclear | Yes | Yes | Korea |
| 1. ^71^Swank 2011 | Unclear | No | Unclear | No | No | Unclear | Yes | Yes | Yes | Yes | United States |
| 1. ^72^Takacs 2017 | Yes | No | Yes | No | No | Yes | Yes | No | Yes | Yes | Canada |
| 1. ^73^Teirlinck 2016 | Yes | Yes | Yes | No | No | No | Yes | Yes | Yes | Yes | Netherlands |
| 1. ^74^Thamalikitkul 2002 | Unclear | Yes | Unclear | No | No | Unclear | Unclear | Unclear | Yes | Yes | Thailand |
| 1. ^75^Thorstensson 2005 | Yes | No | Yes | No | No | Unclear | Unclear | Unclear | Yes | Yes | Sweden |
| 1. ^76^Topp 2002 | Unclear | No | Unclear | No | No | Unclear | Unclear | Unclear | Yes | Yes | United States |
| 1. ^77^Topp 2009 | Unclear | No | Unclear | No | No | Unclear | Unclear | Unclear | Yes | Yes | United States |
| 1. ^78^Van Baar 1998 | Yes | Yes | Yes | No | No | Yes | Yes | Yes | Yes | Yes | Netherland |
| 1. ^80^Wallis 2017 | Yes | No | Yes | No | No | Yes | Yes | Yes | Yes | Yes | Australia |
| 1. ^81^Wang 2007 | Unclear | No | Unclear | No | No | Unclear | No | Yes | Yes | Yes | Taiwan |
| 1. ^82^Wang 2011 | Yes | No | Unclear | No | No | Yes | No | No | Yes | Yes | Taiwan |
| 1. ^83^Weidenhielm 1993 | Yes | No | Unclear | No | No | Unclear | Yes | Unclear | Yes | Yes | Sweden |
| 1. ^84^Wortley 2013 | Unclear | No | Unclear | No | No | Unclear | No | No | Unclear | Yes | United States |

1. Abbott JH, Robertson MC, Chapple C, et al. Manual therapy, exercise therapy, or both, in addition to usual care, for osteoarthritis of the hip or knee: a randomized controlled trial. 1: clinical effectiveness. *Osteoarthritis and Cartilage* 2013; 21(4): 525-34.

2. Aglamis B, Toraman NF, Yaman H. The effect of a 12-week supervised multicomponent exercise program on knee OA in Turkish women. *Journal of Back & Musculoskeletal Rehabilitation* 2008; 21(2): 121-8.

3. Ağlamış B, Toraman NF, Yaman H. Change of quality of life due to exercise training in knee osteoarthritis: SF-36 and Womac. *Journal of Back & Musculoskeletal Rehabilitation* 2009; 22(1): 43-8.

4. An B, Dai K, Zhu Z, et al. Baduanjin alleviates the symptoms of knee osteoarthritis. *Journal of alternative and complementary medicine* 2008; 14(2): 167-74.

5. Aoki O, Tsumura N, Kimura A, Okuyama S, Takikawa S, Hirata S. Home stretching exercise is effective for improving knee range of motion and gait in patients with knee osteoarthritis. *Journal of Physical Therapy Science* 2009; 21(2): 113-119.

6. Arnold CM, Faulkner RA. The effect of aquatic exercise and education on lowering fall risk in older adults with hip osteoarthritis [corrected] [published erratum appears in J AGING PHYS ACTIVITY 2010 Oct;18(4):477-479]. *Journal of Aging & Physical Activity* 2010; 18(3): 245-60.

7. Beaupre LA, Lier D, Davies DM, Johnston DB. The effect of a preoperative exercise and education program on functional recovery, health related quality of life, and health service utilization following primary total knee arthroplasty. *Journal of Rheumatology* 2004; 31(6): 1166-73.

8. Bennell KL, Hunt MA, Wrigley TV, et al. Hip strengthening reduces symptoms but not knee load in people with medial knee osteoarthritis and varus malalignment: a randomised controlled trial. *Osteoarthritis and Cartilage* 2010; 18(5): 621-8.

9. Börjesson M, Robertson E, Weidenhielm L, Mattsson E, Olsson E. Physiotherapy in knee osteoarthrosis: effect on pain and walking. *Physiotherapy Research International* 1996; 1(2): 89-97

10. Braghin RdMB, Libardi EC, Junqueira C, Nogueira–Barbosa MH, de Abreu DCC. Exercise on balance and function for knee osteoarthritis: A randomized controlled trial. *Journal of Bodywork and Movement Therapies* 2018; 22(1): 76-82.

11. Bruce-Brand RA, Walls RJ, Ong JC, Emerson BS, O'Byrne JM, Moyna NM. Effects of home-based resistance training and neuromuscular electrical stimulation in knee osteoarthritis: a randomized controlled trial. *BMC Musculoskeletal Disorders* 2012; 13(1): 118.

12. Calatayud J, Casaña J, Ezzatvar Y, Jakobsen MD, Sundstrup E, Andersen LL. High-intensity preoperative training improves physical and functional recovery in the early post-operative periods after total knee arthroplasty: a randomized controlled trial. *Knee Surgery, Sports Traumatology, Arthroscopy* 2017; 25(9): 2864-72.

13. Chen TW, Lin CW, Lee CL, et al. The efficacy of shock wave therapy in patients with knee osteoarthritis and popliteal cyamella. *The Kaohsiung Journal of Medical Sciences* 2014; 30(7): 362-370.

14. Chopp-Hurley JN, Brenneman EC, Wiebenga EG, Bulbrook B, Keir PJ, Maly MR. Randomized controlled trial investigating the role of exercise in the workplace to improve work ability, performance, and patient-reported symptoms among older workers with osteoarthritis. *Journal of Occupational and Environmental Medicine* 2017; 59(6): 550-6.

15. Christensen R, Henriksen M, Leeds AR, et al. Effect of weight maintenance on symptoms of knee osteoarthritis in obese patients: a twelve-month randomized controlled trial [with consumer summary]. *Arthritis Care & Research* 2015; 67(5): 640-650.

16. Henriksen M, Christensen R, Hunter DJ, et al. Structural changes in the knee during weight loss maintenance after a significant weight loss in obese patients with osteoarthritis: a report of secondary outcome analyses from a randomized controlled trial. *Osteoarthritis and Cartilage* 2014;22(5): 639-646.

17. Cochrane T, Davey RC, Matthes Edwards SM. Randomised controlled trial of the cost-effectiveness of water-based therapy for lower limb osteoarthritis. *Health Technology Assessment (Winchester, England)* 2005;9(31):1-130.

18. Cheung C, Wyman JF, Resnick B, Savik K. Yoga for managing knee osteoarthritis in older women: a pilot randomized controlled trial. *BMC Complementary and Alternative Medicine* 2014; 14:160.

19. D'Lima DD, Colwell Jr CW, Morris BA, Hardwick ME, Kozin F. The effect of preoperative exercise on total knee replacement outcomes. *Clinical Orthopaedics and Related Research* 1996; 326: 174-82.

20. Espejo Antunez L, Cardero Duran MA, Caro Puertolas B, Tellez de Peralta G. Efectos del ejercicio fisico en la funcionalidad y calidad de vida en mayores institucionalizados diagnosticados de gonartrosis (Effects of exercise on the function and quality of life in the institutionalised elderly diagnosed with gonarthrosis) [Spanish]. *Revista Espanola de Geriatria y Gerontologia* 2012; 47(6):262-265.

21. Evcik D, Sonel B. Effectiveness of a home-based exercise therapy and walking program on osteoarthritis of the knee. *Rheumatology International* 2002; 22(3):103-106.

22. Evgeniadis G, Beneka A, Malliou P, Mavromoustakos S, Godolias G. Effects of pre- or postoperative therapeutic exercise on the quality of life, before and after total knee arthroplasty for osteoarthritis. *Journal of Back and Musculoskeletal Rehabilitation* 2008; 21(3): 161-169.

23. Ferrara PE, Rabini A, Maggi L, et al. Effect of pre-operative physiotherapy in patients with end-stage osteoarthritis undergoing hip arthroplasty.[Erratum appears in Clin Rehabil. 2008 Dec;22(12):1137 Note: Lombi, G Magliocchetti [corrected to Magliocchetti, G]]. *Clinical Rehabilitation* 2008; 22(10-11): 977-86.

24. Fransen M, Crosbie J, Edmonds J. Physical therapy is effective for patients with osteoarthritis of the knee: a randomized controlled clinical trial. *Journal of rheumatology*, 2001; 28(1): 156-64.

25. Fransen M, Nairn L, Winstanley J, Lam P, Edmonds J. Physical activity for osteoarthritis management: A randomized controlled clinical trial evaluating hydrotherapy or Tai Chi classes. *Arthritis & Rheumatism-Arthritis Care & Research* 2007; 57(3): 407-14.

26. French HP, Cusack T, Brennan A, et al. Exercise and manual physiotherapy arthritis research trial (EMPART) for osteoarthritis of the hip: a multicenter randomized controlled trial.[Erratum appears in Arch Phys Med Rehabil. 2013 Mar;94(3):600 Note: Fitzpatrick, Martina [added]]. *Archives of Physical Medicine & Rehabilitation* 2013; 94(2): 302-14.

27. Ghroubi S, Elleuch H, Kaffel N, Echikh T, Abid M, Elleuch MH. Contribution of exercise and diet in the management of knee osteoarthritis in the obese. *Annales de Readaptation et de Medecine Physique* 2008; 51(8): 663-70.

28. Gur H, Cakin N, Akova B, Okay E, Kucukoglu S. Concentric versus combined concentric-eccentric isokinetic training: effects on functional capacity and symptoms in patients with osteoarthrosis of the knee. *Archives of Physical Medicine and Rehabilitation* 2002; 83(3): 308-16.

29. Hasegawa R, Islam MM, Nasu E, et al. Effects of Combined Balance and Resistance Exercise on Reducing Knee Pain in Community-Dwelling Older Adults. *Physical & Occupational Therapy in Geriatrics* 2010; 28(1): 44-56.

30. Henriksen M, Klokker L, Graven-Nielsen T, et al. Association of exercise therapy and reduction of pain sensitivity in patients with knee osteoarthritis: a randomized controlled trial. *Arthritis Care & Research* 2014; 66(12): 1836-43.

31. Henriksen M, Klokker L, Bartholdy C, Schjoedt-Jorgensen T, Bandak E, Bliddal H. No effects of functional exercise therapy on walking biomechanics in patients with knee osteoarthritis: exploratory outcome analyses from a randomised trial. *BMJ Open Sport & Exercise Medicine* 2017; 2(1): bmjsem-2017-000230.

32. Hermann A, Holsgaard-Larsen A, Zerahn B, Mejdahl S, Overgaard S. Preoperative progressive explosive-type resistance training is feasible and effective in patients with hip osteoarthritis scheduled for total hip arthroplasty -- a randomized controlled trial. *Osteoarthritis and Cartilage* 2016; 24(1):91-98.

33. Hinman RS, Heywood SE, Day AR. Aquatic physical therapy for hip and knee osteoarthritis: results of a single-blind randomized controlled trial. *Physical Therapy* 2007; 87(1): 32-43.

34. Hoogeboom TJ, Dronkers JJ, van den Ende CHM, Oosting E, van Meeteren NLU. Preoperative therapeutic exercise in frail elderly scheduled for total hip replacement: a randomized pilot trial [with consumer summary]. *Clinical Rehabilitation* 2010; 24(10): 901-910.

35. Huang MH, Lin YS, Yang RC, Lee CL. A comparison of various therapeutic exercises on the functional status of patients with knee osteoarthritis. *Seminars in Arthritis and Rheumatism* 2003; 32(6): 398-406.

36. Huang MH, Yang RC, Lee CL, Chen TW, Wang MC. Preliminary results of integrated therapy for patients with knee osteoarthritis. *Arthritis Care & Research* 2005; 53(6): 812-820.

37. Huang L, Guo B, Xu F, Zhao J. Effects of quadriceps functional exercise with isometric contraction in the treatment of knee osteoarthritis. *International Journal of Rheumatic Diseases* 2018; 21(5): 952-9.

38. Hunt MA, Pollock CL, Kraus VB, et al. Relationships amongst osteoarthritis biomarkers, dynamic knee joint load, and exercise: results from a randomized controlled pilot study. *BMC Musculoskeletal Disorders* 2013 27; 14(1): 115.

39. Jan M-H, Lin J-J, Liau J-J, Lin Y-F, Lin D-H. Investigation of clinical effects of high- and low-resistance training for patients with knee osteoarthritis: A randomized controlled trial. *Physical Therapy* 2008; 88(4): 427-36.

40. Jan M-H, Lin C-H, Lin Y-F, Lill J-J, Lin D-H. Effects of Weight-Bearing Versus Nonweight-Bearing Exercise on Function, Walking Speed, and Position Sense in Participants With Knee Osteoarthritis: A Randomized Controlled Trial. *Archives of Physical Medicine and Rehabilitation* 2009; 90(6): 897-904.

41. Jorge RTB, Souza MCd, Chiari A, et al. Progressive resistance exercise in women with osteoarthritis of the knee: a randomized controlled trial. *Clinical Rehabilitation* 2015; 29(3): 234-43.

42. Juhakoski R, Tenhonen S, Malmivaara A, Kiviniemi V, Anttonen T, Arokoski JP. A pragmatic randomized controlled study of the effectiveness and cost consequences of exercise therapy in hip osteoarthritis [with consumer summary]. *Clinical Rehabilitation* 2011; 25(4): 370-383.

43. Koli J, Multanen J, Kujala UM, et al. Effects of exercise on patellar cartilage in women with mild knee osteoarthritis. *Medicine and Science in Sports and Exercise* 2015; 47(9): 1767-1774.

44. Krasilshchikov O, Sungkit NB, Shihabudin TM, Shaw I, Shaw BS. Effects of an eight-week training programme on pain relief and physical condition of overweight and obese women with early stage primary knee osteoarthritis. *African Journal for Physical, Health Education, Recreation and Dance* 2011; 17(2): 328-339.

45. Krauss I, Steinhilber B, Haupt G, Miller R, Martus P, Janssen P. Exercise therapy in hip osteoarthritis-a randomized controlled trial. *Deutsches Arzteblatt International* 2014; 111(35-36): 592.

46. Steinhilber B, Haupt G, Miller R, Janssen P, Krauss I. Exercise therapy in patients with hip osteoarthritis: effect on hip muscle strength and safety aspects of exercise -- results of a randomized controlled trial. *Modern Rheumatology* 2017; 27(3): 493-502.

47. Kreindler H, Lewis CB, Rush S, Schaefer K. Effects of three exercise protocols on strength of persons with osteoarthritis of the knee. *Topics in Geriatric Rehabilitation* 1989; 4(3): 32-39.

48. Lee HY, Lee KJ. [Effects of Tai Chi exercise in elderly with knee osteoarthritis]. *Daehan Ganho Haghoeji* 2008; 38(1): 11-8.

49. Lee H-J, Park H-J, Chae Y, et al. Tai Chi Qigong for the quality of life of patients with knee osteoarthritis: a pilot, randomized, waiting list controlled trial. *Clinical Rehabilitation* 2009; 23(6): 504-11.

50. Lim B-W, Hinman RS, Wrigley TV, Sharma L, Bennell KL. Does knee malalignment mediate the effects of quadriceps strengthening on knee adduction moment, pain, and function in medial knee osteoarthritis? A randomized controlled trial. *Arthritis & Rheumatism-Arthritis Care & Research* 2008; 59(7): 943-51.

51. Lin D-H, Lin C-HJ, Lin Y-F, Jan M-H. Efficacy of 2 Non-Weight-Bearing Interventions, Proprioception Training Versus Strength Training, for Patients With Knee Osteoarthritis: A Randomized Clinical Trial. *Journal of Orthopaedic & Sports Physical Therapy* 2009; 39(6): 450-7.

52. Lund H, Weile U, Christensen R, et al. A randomized controlled trial of aquatic and land-based exercise in patients with knee osteoarthritis. *Journal of Rehabilitation Medicine (Stiftelsen Rehabiliteringsinformation)* 2008; 40(2): 137-44.

53. Moghadam EB, Shojaedin SS. The effect of eight weeks aerobic training on functional indicators and range of motion in active older men with knee osteoarthritis. *Razi Journal of Medical Sciences* 2017; 24(156): 100-10.

54. Munukka M, Waller B, Rantalainen T, et al. Efficacy of progressive aquatic resistance training for tibiofemoral cartilage in postmenopausal women with mild knee osteoarthritis: a randomised controlled trial. *Osteoarthritis and Cartilage 2*016; 24(10): 1708-1717.

55. Waller B, Munukka M, Rantalainen T, et al. Effects of high intensity resistance aquatic training on body composition and walking speed in women with mild knee osteoarthritis: a 4-month RCT with 12-month follow-up. *Osteoarthritis and Cartilage* 2017; 25(8): 1238-46.

56. Oida Y, Morozumi K, Nakamura N, et al. (Effectiveness of a community health service program using exercise intervention for elderly people with osteoarthritis of the knees: a randomized controlled trial) [Japanese]. *Nippon Koshu Eisei Zasshi [Japanese Journal of Public Health]* 2008; 55(4):228-237.

57. Oosting E, Jans MP, Dronkers JJ, et al. Preoperative Home-Based Physical Therapy Versus Usual Care to Improve Functional Health of Frail Older Adults Scheduled for Elective Total Hip Arthroplasty: A Pilot Randomized Controlled Trial. *Archives of Physical Medicine & Rehabilitation* 2012; 93(4): 610-6.

58. O'Reilly SC, Muir KR, Doherty M. Effectiveness of home exercise on pain and disability from osteoarthritis of the knee: a randomised controlled trial. *Annals of the Rheumatic Diseases* 1999; 58(1): 15-19.

59. Rapp W, Boeer J, Albrich C, Heitkamp HC. Efficiency of Vibration or Strength Training for Knee Stability in Osteoarthritis of the Knee. [German]

Auswirkung eines Vibrations- und Krafttrainings auf die Beinmuskulatur bei Gonarthrosepatienten. *Aktuelle Rheumatologie* 2009; 34(4): 240-5.

60. Rogind H, Bibow-Nielsen B, Jensen B, Moller HC, Frimodt-Moller H, Bliddal H. The effects of a physical training program on patients with osteoarthritis of the knees. *Archives of Physical Medicine and Rehabilitation* 1998; 79(11): 1421-7.

61. de Rooij M, van der Leeden M, Cheung J, et al. Efficacy of tailored exercise therapy on physical functioning in patients with knee osteoarthritis and comorbidity: a randomized controlled trial [with consumer summary]. *Arthritis Care & Research* 2017; 69(6): 807-16 62. Rosedale R, Rastogi R, May S, et al. Efficacy of exercise intervention as determined by the McKenzie system of mechanical diagnosis and therapy for knee osteoarthritis: a randomized controlled trial [with consumer summary]. *The Journal of Orthopaedic and Sports Physical Therapy* 2014; 44(3): 173-81.

63. Salacinski AJ, Krohn K, Lewis SF, Holland ML, Ireland K, Marchetti G. The Effects of Group Cycling on Gait and Pain-Related Disability in Individuals With Mild-to-Moderate Knee Osteoarthritis: A Randomized Controlled Trial. *Journal of Orthopaedic & Sports Physical Therapy* 2012; 42(12): 985-95.

64. Salli A, Sahin N, Baskent A, Ugurlu H. The effect of two exercise programs on various functional outcome measures in patients with osteoarthritis of the knee: A randomized controlled clinical trial. *Isokinetics and Exercise Science* 2010; 18(4): 201-9.

65. Salli A, Ugurlu H, Emlik D. Comparison of the effectiveness of concentric, combined concentric-eccentric and isometric exercises on symptoms and functional capacity in patients with knee osteoarthritis. [Turkish]*Turkiye Fiziksel Tip ve Rehabilitasyon Dergisi* 2006; 52(2): 61-7.

66. Schilke JM, Johnson GO, Housh TJ, Odell JR. Effects of muscle strength training on the functional status of patients with osteoarthritis of the knee joint. *Nursing Research* 1996; 45(2): 68-72.

67. Sekir U, Gur H. A multi-station proprioceptive exercise program in patients with bilateral knee osteoarthrosis: functional capacity, pain and sensoriomotor function. A randomized controlled trial [with consumer summary]. *Journal of Sports Science and Medicine* 2005; 4(4):590-603.

68. Simao AP, Avelar NC, Tossige-Gomes R, et al. Functional Performance and Inflammatory Cytokines After Squat Exercises and Whole-Body Vibration in Elderly Individuals With Knee Osteoarthritis. *Archives of Physical Medicine and Rehabilitation* 2012; 93(10): 1692-700.

69. Skoffer B, Maribo T, Mechlenburg I, Hansen PM, Søballe K, Dalgas U. Efficacy of preoperative progressive resistance training on postoperative outcomes in patients undergoing total knee arthroplasty. *Arthritis Care & Research* 2016; 68(9): 1239-51.

70. Sung-Bum JU, Gi Duck P, Sang-Soo KIM. Effects of proprioceptive circuit exercise on knee joint pain and muscle function in patients with knee osteoarthritis. *Journal of Physical Therapy Science* 2015; 27(8): 2439-41.

71. Swank AM, Kachelman JB, Bibeau W, et al. Prehabilitation before total knee arthroplasty increases strength and function in older adults with severe osteoarthritis [with consumer summary]. *Journal of Strength & Conditioning Research* 2011; 25(2): 318-25.

72. Takacs J, Krowchuk NM, Garland SJ, Carpenter MG, Hunt MA. Dynamic balance training improves physical function in individuals with knee osteoarthritis: A pilot randomized controlled trial. *Archives of Physical Medicine and Rehabilitation* 2017; 98(8): 1586-93.

73. Teirlinck CH, Luijsterburg PAJ, Dekker J, et al. Effectiveness of exercise therapy added to general practitioner care in patients with hip osteoarthritis: a pragmatic randomized controlled trial. *Osteoarthritis and Cartilage* 2016; 24(1): 82-90.

74. Kuptniratsaikul V, Tosayanonda O, Nilganuwong S, Thamalikitkul V. The efficacy of a muscle exercise program to improve functional performance of the knee in patients with osteoarthritis. *Chotmaihet Thangphaet [Journal of the Medical Association of Thailand]* 2002; 85(1): 33-40.

75. Thorstensson CA, Roos EM, Petersson IF, Ekdahl C. Six-week high-intensity exercise program for middle-aged patients with knee osteoarthritis: a randomized controlled trial. *BMC Musculoskeletal Disorders* 2005; 6(1): 27.

76. Topp R, Woolley S, Hornyak J, III, Khuder S, Kahaleh B. The effect of dynamic versus isometric resistance training on pain and functioning among adults with osteoarthritis of the knee. *Archives of Physical Medicine and Rehabilitation* 2002; 83(9): 1187-95.

77. Topp R, Swank AM, Quesada PM, Nyland J, Malkani A. The effect of prehabilitation exercise on strength and functioning after total knee arthroplasty. *PM&R* 2009; 1(8): 729-735.

78. Van Baar ME, Dekker J, Oostendorp RAB, et al. The effectiveness of exercise therapy in patients with osteoarthritis of the hip or knee: A randomized clinical trial. *Journal of Rheumatology* 1998; 25(12): 2432-9.

79. van Baar ME, Dekker J, Oostendorp RAB, Bijl D, Voorn TB, Bijlsma JWJ. Effectiveness of exercise in patients with osteoarthritis of hip or knee: nine months' follow up. *Annals of the Rheumatic Diseases* 2001; 60(12): 1123-30.

80. Wallis J, Webster K, Levinger P, Singh P, Fong C, Taylor N. A walking program for people with severe knee osteoarthritis did not reduce pain but may have benefits for cardiovascular health: a phase II randomised controlled trial. *Osteoarthritis and Cartilage* 2017; 25(12): 1969-79.

81. Wang T-J, Belza B, Elaine Thompson F, Whitney JD, Bennett K. Effects of aquatic exercise on flexibility, strength and aerobic fitness in adults with osteoarthritis of the hip or knee. *Journal of Advanced Nursing* 2007; 57(2): 141-52.

82. Wang TJ, Lee SC, Liang SY, Tung HH, Wu SF, Lin YP. Comparing the efficacy of aquatic exercises and land-based exercises for patients with knee osteoarthritis. *Journal of Clinical Nursing* 2011; 20(17-18): 2609-2622.

83. Weidenhielm L, Mattsson E, Broström LA, Wersäll-Robertsson E. Effect of preoperative physiotherapy in unicompartmental prosthetic knee replacement. *Scandinavian Journal of Rehabilitation Medicine* 1993; 25(1): 33-9.

84. Wortley M, Zhang S, Paquette M, et al. Effects of resistance and Tai Ji training on mobility and symptoms in knee osteoarthritis patients. *Journal of Sport and Health Science* 2013; 2(4): 209-214.

**Appendix 3.** Funnel plots for all outcomes for exercise versus usual care.


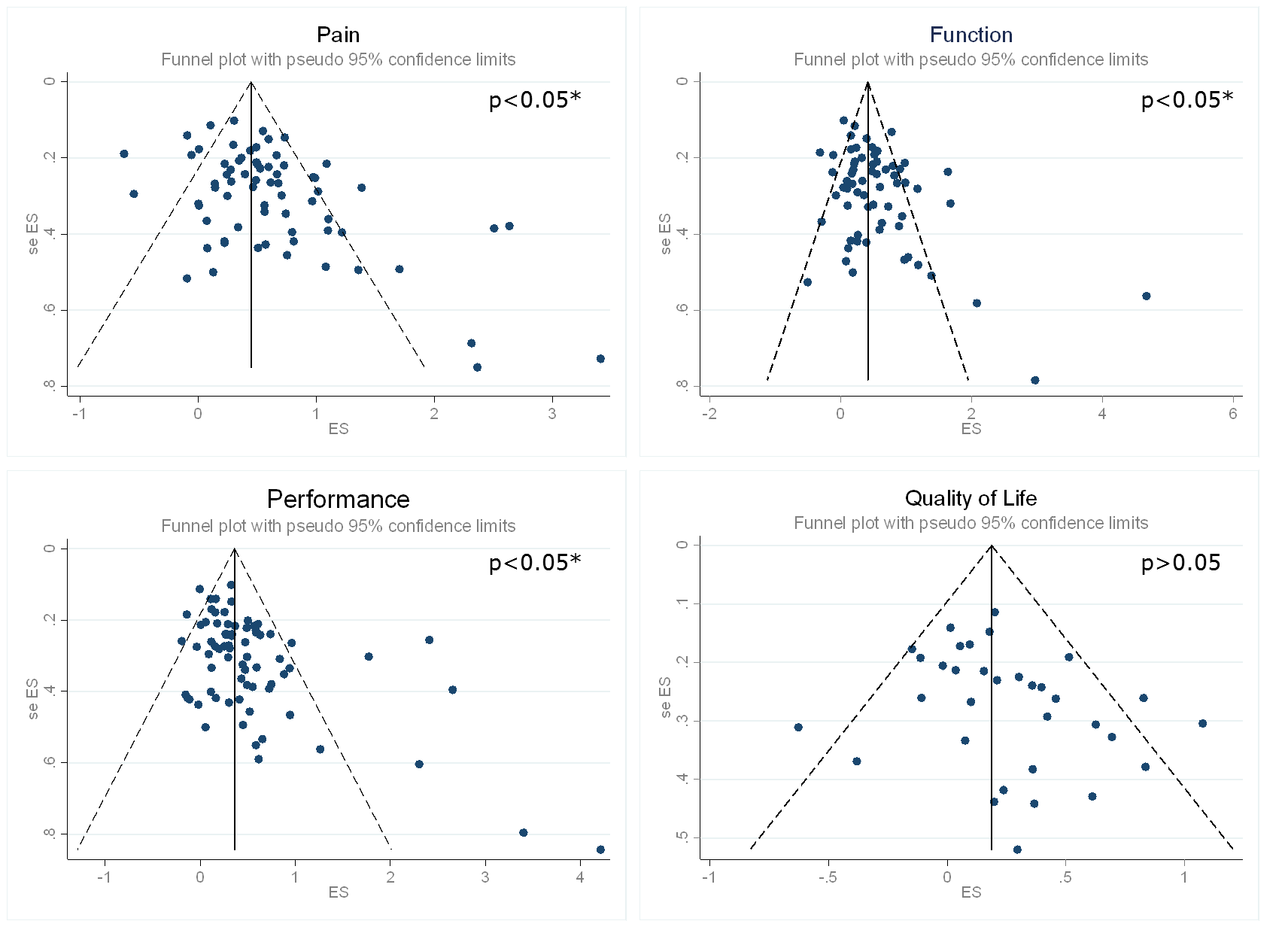


Plot to assess the bias/symmetrical distribution in evidence.

* Egger’s test suggestive of publication bias at p<0.05.

**Appendix 4.** Risk of bias.

* Only studies with performance measures were scored.

ITT, intention-to-treat

**Appendix 5.** Overall effect of exercise versus usual care.

A. Function

ES, effect size; 95% CI, 95% confidence interval

B. Performance

C. Quality of life

**Appendix 6.** Subgroup analysis.

A**.** Function

|  | **No. comp. (No. trials)** | **No. Patients** | **Effect Size (95%CI)** | **I^2^ (%)** | **p-value** | |
| --- | --- | --- | --- | --- | --- | --- |
|  |  |  |  |  | **Univariate** | **Multivariate** |
| **Overall** | **65 (64)** | 5025 | 0.51 (0.38–0.63) | 74.5 |  |  |
| **Age, years** |  |  |  |  |  |  |
| <60 | 12 | 591 | 1.18 (0.65–1.72) | 87.3 | <0.01* | <0.01* |
| ≥60 | 53 | 4434 | 0.41 (0.30–0.52) | 64.6 |  |  |
| **% Female^a^** | |  |  |  |  |  |
| <60% | 12 | 1023 | 0.24 (0.07–0.41) | 38.2 | 0.14 | - |
| ≥60% | 29 | 2588 | 0.52 (0.35–0.69) | 74.4 |  |  |
| ≥80% | 21 | 1320 | 0.49 (0.31–0.68) | 57.6 |  |  |
| **Mean BMI, kg/m^2 a^** | |  |  |  |  |  |
| <30 | 26 | 2111 | 0.41 (0.27–0.54) | 47.5 | 0.77 | - |
| ≥30 | 17 | 1103 | 0.51 (0.21–0.81) | 81.1 |  |  |
| **Joint** |  |  |  |  |  |  |
| Knee | 52 | 4443 | 0.59 (0.44–0.74) | 76.9 | 0.07* | 0.31 |
| Hip | 8 | 703 | 0.21 (-0.03–0.46) | 57.8 |  |  |
| Mixed | 5 | 611 | 0.31 (0.15–0.47) | 0.0 |  |  |
| **On TJR waiting list** | |  |  |  |  |  |
| No | 54 | 4443 | 0.52 (0.40–0.64) | 69.5 | 0.54 | - |
| Yes | 11 | 582 | 0.45 (-0.04–0.95) | 86.5 |  |  |
| **“Explicit” pain criteria** | |  |  |  |  |  |
| None | 51 | 3758 | 0.50 (0.36–0.65) | 75.2 | 0.89 | - |
| Yes | 14 | 1267 | 0.53 (0.30–0.75) | 72.4 |  |  |
| **ACR criteria** |  |  |  |  |  |  |
| Yes | 32 | 2493 | 0.62 (0.43–0.81) | 79.4 | 0.19 | - |
| No/unclear | 33 | 2532 | 0.38 (0.24–0.52) | 61.0 |  |  |
| **Radiographic requirement** | |  |  |  |  |  |
| Yes | 38 | 2686 | 0.61 (0.42–0.81) | 81.3 | 0.20 | - |
| No/unclear | 27 | 2339 | 0.38 (0.25–0.51) | 49.1 |  |  |
| **Adherence monitored** | |  |  |  |  |  |
| Yes | 43 | 3765 | 0.35 (0.24–0.46) | 62.0 | <0.01* | <0.01* |
| No/unclear | 22 | 1260 | 0.88 (0.60–1.15) | 78.0 |  |  |
| **Analgesic control/monitored** | |  |  |  |  |  |
| Yes | 31 | 2674 | 0.49 (0.33–0.66) | 74.5 | 0.92 | - |
| No/ unclear | 34 | 2351 | 0.52 (0.34–0.70) | 74.8 |  |  |
| **Recruitment centre^a^** | |  |  |  |  |  |
| Specialist/hospital | 24 | 1658 | 0.52 (0.28–0.76) | 79.7 | 0.33 | - |
| GP/community | 25 | 3007 | 0.28 (0.16–0.41) | 41.9 |  |  |
| Mixed | 7 | 680 | 0.46 (0.31–0.61) | 0.0 |  |  |
| **ITT use** |  |  |  |  |  |  |
| Yes | 41 | 3337 | 0.47 (0.32–0.62) | 76.4 | 0.50 | - |
| No/unclear | 24 | 1688 | 0.58 (0.37–0.79) | 71.0 |  |  |
| **>100/group** |  |  |  |  |  |  |
| Yes | 4 | 1148 | 0.29 (-0.02–0.60) | 85.6 | 0.41 | - |
| No | 61 | 3877 | 0.53 (0.40–0.66) | 72.8 |  |  |

95% CI, 95% confidence interval; No. comp., number of comparators; BMI, body mass index; ACR, American College of Rheumatology; GP, general practitioner; ITT, intent-to-treat; OA, osteoarthritis; TJR, total joint replacement

* significant at p≤0.10; ^a^Data were missing in some studies

B. Performance

|  |  | **No. comp. (No. trials)** | **No. Patients** | **Effect Size (95%CI)** | **I^2^ (%)** | **p-value** | |
| --- | --- | --- | --- | --- | --- | --- | --- |
|  |  |  |  |  |  | **Univariate** | **Multivariate** |
| **Overall** |  | **73 (72)** | **5082** | **0.46 (0.35–0.58)** | 70.5 |  |  |
| **Age, years** |  |  |  |  |  |  |  |
| <60 | | 14 | 613 | 0.81 (0.37–1.26) | 83.1 | 0.09* | 0.18 |
| ≥60 | | 59 | 4469 | 0.41 (0.30–0.52) | 64.3 |  |  |
| **% Female^a^** | | |  |  |  |  |  |
| <60% | | 14 | 993 | 0.29 (0.16–0.41) | 0.0 | 0.18 | - |
| ≥60% | | 33 | 2863 | 0.37 (0.22–0.52) | 70.2 |  |  |
| ≥80% | | 22 | 1114 | 0.50 (0.31–0.68) | 50.3 |  |  |
| **Mean BMI, kg/m^2 a^** | | |  |  |  |  |  |
| <30 | | 30 | 1943 | 0.37 (0.24–0.50) | 41.1 | 0.91 | - |
| ≥30 | | 18 | 1078 | 0.46 (0.19–0.72) | 76.2 |  |  |
| **Joint** | |  |  |  |  |  |  |
| Knee | | 57 | 3484 | 0.56 (0.41–0.70) | 71.9 | 0.04* | 0.70 |
| Hip | | 9 | 753 | 0.17 (0.00–0.33) | 16.9 |  |  |
| Mixed | | 7 | 845 | 0.17 (-0.00–0.34) | 25.9 |  |  |
| **On TJR waiting list** | | |  |  |  |  |  |
| No | | 59 | 4400 | 0.45 (0.32–0.57) | 71.5 | 0.68 | - |
| Yes | | 14 | 682 | 0.53 (0.25–0.80) | 66.4 |  |  |
| **“Explicit” pain criteria** | | |  |  |  |  |  |
| None | | 58 | 3775 | 0.46 (0.33–0.60) | 73.3 | 0.89 | - |
| Yes | | 15 | 1307 | 0.46 (0.25–0.66) | 63.3 |  |  |
| **ACR criteria** | |  |  |  |  |  |  |
| Yes | | 36 | 2488 | 0.45 (0.25–0.64) | 80.2 | 0.67 | - |
| No/unclear | | 37 | 2594 | 0.45 (0.33–0.57) | 46.1 |  |  |
| **Radiographic requirement** | | |  |  |  |  |  |
| Yes | | 40 | 2586 | 0.57 (0.39–0.75) | 75.7 | 0.14 | - |
| No/unclear | | 33 | 2496 | 0.33 (0.20–0.46) | 54.3 |  |  |
| **Adherence monitored** | | |  |  |  |  |  |
| Yes | | 48 | 3985 | 0.32 (0.21–0.43) | 60.7 | <0.01* | 0.58 |
| No/unclear | | 25 | 1097 | 0.81 (0.54–1.08) | 74.4 |  |  |
| **Analgesic control/monitored** | | |  |  |  |  |  |
| Yes | | 34 | 2924 | 0.32 (0.21–0.43) | 45.4 | 0.09* | 0.63 |
| No/ unclear | | 39 | 2158 | 0.60 (0.39–0.81) | 78.3 |  |  |
| **Recruitment centre^a^** | | |  |  |  |  |  |
| Specialist/hospital | | 26 | 1468 | 0.67 (0.41–0.93) | 79.4 | <0.01* | 0.04* |
| GP/community | | 29 | 2524 | 0.25 (0.16–0.34) | 11.4 |  |  |
| Mixed | | 7 | 676 | 0.17 (0.02–0.32) | 0.0 |  |  |
| **ITT use** |  |  |  |  |  |  |  |
| Yes | | 46 | 3559 | 0.34 (0.24–0.44) | 52.0 | 0.04* | 0.4 |
| No/unclear | | 27 | 1523 | 0.70 (0.43–0.96) | 80.2 |  |  |
| **>100/ group** |  |  |  |  |  |  |  |
| Yes | | 4 | 1108 | 0.16 (0.00–0.31) | 38.5 | 0.19 | - |
| No | | 69 | 3974 | 0.49 (0.37–0.62) | 69.8 |  |  |
| **Performance measure** | | |  |  |  |  |  |
| Walking | | 37 | 2783 | 0.47 (0.29–0.65) | 78.6 | 0.91 | - |
| Others | | 36 | 2299 | 0.45 (0.32–0.59) | 53.6 |  |  |

95% CI, 95% confidence interval; No. comp., number of comparators; BMI, body mass index; ACR, American College of Rheumatology; GP, general practitioner; ITT, intent-to-treat; OA, osteoarthritis; TJR, total joint replacement

C**.** Quality of life (QoL)

|  | **No. comp. (No. trials)** | **No. Patients** | **Effect Size (95%CI)** | **I^2^ (%)** | **p-value** | |
| --- | --- | --- | --- | --- | --- | --- |
|  |  |  |  |  | **Univariate** | **Multivariate** |
| **Overall** | 33 | 2629 | 0.21 (0.11–0.31) | 36.4 |  |  |
| **Age, years** |  |  |  |  |  |  |
| <60 | 7 | 446 | 0.39 (0.10–0.69) | 52.6 | 0.06* | 0.23 |
| ≥60 | 26 | 2183 | 0.17 (0.06–0.27) | 27.8 |  |  |
| **% Female^a^** | |  |  |  |  |  |
| <60% | 11 | 707 | 0.20 (0.03–0.37) | 10.5 | 0.91 | - |
| ≥60% | 14 | 1239 | 0.24 (0.07–0.40) | 43.8 |  |  |
| ≥80% | 7 | 633 | 0.07 (-0.08–0.22) | 0.0 |  |  |
| **Mean BMI, kg/m^2 a^** | |  |  |  |  |  |
| <30 | 15 | 1339 | 0.19 (0.09–0.30) | 0.0 | 0.84 | - |
| ≥30 | 13 | 913 | 0.20 (-0.03–0.43) | 64.4 |  |  |
| **Joint** |  |  |  |  |  |  |
| Knee | 23 | 1567 | 0.27 (0.12–0.41) | 47.9 | 0.33 | - |
| Hip | 7 | 585 | 0.08 (-0.08–0.25) | 0.0 |  |  |
| Mixed | 3 | 477 | 0.18 (-0.01–0.36) | 0.0 |  |  |
| **On TJR waiting list** | |  |  |  |  |  |
| No | 23 | 2059 | 0.38 (0.18–0.57) | 76.7 | 0.31 | - |
| Yes | 10 | 570 | 0.10 (-0.18–0.38) | 58.7 |  |  |
| **“Explicit” pain criteria** | |  |  |  |  |  |
| None | 25 | 1823 | 0.20 (0.06–0.33) | 42.2 | 0.85 | - |
| Yes | 8 | 806 | 0.24 (0.08–0.40) | 17.3 |  |  |
| **ACR criteria** |  |  |  |  |  |  |
| Yes | 13 | 937 | 0.26 (0.04–0.47) | 59.5 | 0.74 | - |
| No/unclear | 20 | 1692 | 0.19 (0.08–0.29) | 8.3 |  |  |
| **Radiographic requirement** | |  |  |  |  |  |
| Yes | 18 | 1200 | 0.26 (0.09–0.44) | 52.6 | 0.38 | - |
| No/unclear | 15 | 1429 | 0.15 (0.05–0.26) | 0.0 |  |  |
| **Adherence monitored** | |  |  |  |  |  |
| Yes | 25 | 2207 | 0.15 (0.07–0.24) | 0.0 | 0.57 | - |
| No/unclear | 8 | 422 | 0.46 (0.06–0.87) | 72.0 |  |  |
| **Analgesic control/monitored** | |  |  |  |  |  |
| Yes | 15 | 1358 | 0.16 (0.03–0.29) | 20.0 | 0.63 | - |
| No/unclear | 18 | 1371 | 0.25 (0.09–0.41) | 46.9 |  |  |
| **Recruitment centre** | |  |  |  |  |  |
| Specialist/hospital | 16 | 1009 | 0.24 (0.02–0.46) | 62.9 | 0.57 | - |
| GP/community | 12 | 1168 | 0.21 (0.10–0.33) | 0.0 |  |  |
| Mixed | 5 | 452 | 0.10 (-0.08–0.29) | 0.0 |  |  |
| **ITT use** |  |  |  |  |  |  |
| Yes | 24 | 2210 | 0.17 (0.05–0.30) | 45.0 | 0.10* | 0.46 |
| No/unclear | 9 | 419 | 0.37 (0.17–0.57) | 0.0 |  |  |
| **>100/ group** |  |  |  |  |  |  |
| Yes | 2 | 512 | 0.13 (-0.06–0.30) | 6.3 | 0.65 | - |
| No | 31 | 2117 | 0.23 (0.11–0.34) | 38.4 |  |  |
| **QoL tool** |  |  |  |  |  |  |
| Disease-specific | 10 | 645 | 0.17 (0.01–0.32) | 0.0 | 0.42 | - |
| Generic | 23 | 1984 | 0.24 (0.10–0.38) | 50.2 |  |  |

95% CI, 95% confidence interval; No. comp., number of comparators; BMI, body mass index; ACR, American College of Rheumatology; GP, general practitioner; ITT, intent-to-treat; OA, osteoarthritis; TJR, total joint replacement

**Appendix 7.** Sensitivity analysis.

|  | **No studies included or excluded** | **No. comparisons (No. trials)** | **No. Patients** | **Effect size**  **(95%CI)** |
| --- | --- | --- | --- | --- |
| 1. **Pain** | | | | |
| **Overall** |  | **69 (68)** | **5272** | **0.56 (0.44–0.68)** |
| Using raw score | - | - | - | 0.59 (0.47–0.71) |
| Outlier: ES>40 | (+1) | 70 | 5343 | 0.60 (0.45–0.74) |
| SD imputed | (-2) | 67 | 4821 | 0.56 (0.44–0.69) |
| Unit (knee) | (-3) | 66 | 5026 | 0.55 (0.43–0.68) |
| Translated | (-5) | 64 | 5150 | 0.55 (0.43–0.67) |
| 1. **Function** | | | | |
| **Overall** |  | **65 (64)** | **5025** | **0.51 (0.38–0.63)** |
| Using raw score | - | - | - | 0.51 (0.38–0.64) |
| Outliers | - | **-** | **-** | **-** |
| SD imputed | (-1) | 64 | 4775 | 0.50 (0.38–0.62) |
| Unit (knee) | (-2) | 62 | 4779 | 0.48 (0.35–0.60) |
| Translated | (-4) | 61 | 4852 | 0.50 (0.38–0.63) |
| 1. **Quality of life** | | | | |
| **Overall** |  | **33** | **2629** | **0.21 (0.11–0.31)** |
| Using raw score | - | - | - | 0.35 (0.17–0.53) |
| Outliers: ES>5 | (+1) | 34 | 2683 | 0.30 (0.14–0.46) |
| SD imputed | - | **-** | **-** | **-** |
| Unit (knee) | - | - | - | - |
| Translated | (-2) | 32 | 2598 | 0.20 (0.10–0.30) |
| 1. **Performance** | | | | |
| **Overall** |  | **73(72)** | **5082** | **0.46 (0.35–0.58)** |
| Using raw score | - | - | - | 0.50 (0.37–0.62) |
| Outliers | - | - | - | - |
| SD imputed | - | - | - | - |
| Unit (knee) | (-3) | 70 | 4836 | 0.39 (0.29–0.48) |
| Translated | (-5) | 68 | 4898 | 0.42 (0.32–0.53) |

The signs “+” and “-” in brackets indicate the number of studies added or removed, respectively, from primary analysis for sensitivity analysis. The overall effect size (ES) estimates were calculated primarily from change score.

SD imputed, studies for which missing SD was imputed; Unit (knee), knee was used as unit of analysis instead of individuals; Translated, translated publication
